# Pterostilbene Alleviates Cholestasis by Promoting SIRT1 Activity in Hepatocytes and Macrophages

**DOI:** 10.3389/fphar.2021.785403

**Published:** 2021-11-25

**Authors:** Chuanrui Ma, Jiaqing Xiang, Guixiao Huang, Yaxi Zhao, Xinyu Wang, Han Wu, Kewei Jiang, Zhen Liang, Lin Kang, Guangyan Yang, Shu Yang

**Affiliations:** ^1^ First Teaching Hospital of Tianjin University of Traditional Chinese Medicine, Tianjin, China; ^2^ National Clinical Research Center for Chinese Medicine Acupuncture and Moxibustion, Tianjin, China; ^3^ Department of Geriatrics, Shenzhen People’s Hospital (The Second Clinical Medical College, Jinan University, The First Affiliated Hospital, Southern University of Science and Technology), Shenzhen, China; ^4^ The 3rd Affiliated Hospital of Shenzhen University, Shenzhen, China; ^5^ Department of Tuberculosis, Shenzhen Third People’s Hospital, Shenzhen, China; ^6^ The Biobank of National Innovation Center for Advanced Medical Devices, Shenzhen People’s Hospital, Shenzhen, China; ^7^ Integrated Chinese and Western Medicine Postdoctoral Research Station, Jinan University, Guangzhou, China

**Keywords:** cholestasis, pterostilbene, SIRT1, FXR, p53

## Abstract

**Background and purpose:** FXR is a promising target for the treatment of human cholestatic liver disease (CLD). SIRT1 is a deacetylase which promotes FXR activity through deacetylating FXR. Pterostilbene (PTE) is an activator of SIRT1. However, the role of PTE in cholestasis has so far not been investigated. We examined whether PTE treatment alleviate liver injury in DDC or ANIT-induced experimental cholestasis, and explored the underlying mechanisms.

**Experimental approach:** Mice with DDC- or ANIT-induced cholestasis were treated with different dose of PTE. Primary hepatocytes and bone marrow derived macrophages were used *in vitro* to assess the molecular mechanism by which PTE may improve CLD. Identical doses of UDCA or PTE were administered to DDC- or ANIT-induced cholestasis mice.

**Key results:** PTE intervention attenuated DDC or ANIT-induced cholestasis. PTE inhibited macrophage infiltration and activation in mouse liver through the SIRT1-p53 signaling pathway, and it improved hepatic bile metabolism through the SIRT1-FXR signaling pathway. Compare with UDCA, the same doses of PTE was more effective in improving cholestatic liver injury caused by DDC or ANIT.

**Conclusion and implications:** SIRT1 activation in macrophages may be an effective CLD treatment avenue. Using CLD models, we thus identified PTE as a novel clinical candidate compound for the treatment of CLD.

## Introduction

Genetic and pathophysiological disruptions in bile acid (BA) metabolism and transport lead to altered bile flow and cholestatic liver injury. Primary biliary cholangitis (PBC) and primary sclerosing cholangitis (PSC) are the two predominant types of chronic cholestatic liver diseases (CLDs) which, if left untreated, result in liver failure (Ali et al., 2015; Dyson et al., 2015). Current recommended therapies for treating cholestasis mainly rely on the use of ursodeoxycholic acid; however, such treatment were not proven to be effective in PSC and are ineffective in a proportion of PBC patients (Hirschfield et al., 2010; Eaton et al., 2013). Therapeutic options for patients with no or limited response to ursodeoxycholic acid are extremely limited. Recent studies suggest that obeticholic acid, a potent synthetic farnesoid X receptor (FXR or NR1H4) agonist, and fibrates, which are PPAR activators, may be of use for such patients (Floreani and Mangini, 2018). Obeticholic acid was recently approved by the United States. Food and Drug Administration for use in PBC patients, whereas fibrates have yet to be tested in randomized trials (Ghonem et al., 2015). Thus, this current limitation regarding therapeutic options emphasizes the urgent need for alternative therapeutic avenues.

FXR, which has been established as the primary BA sensor and a crucial regulator of BA metabolism, plays an important role in the regulation of BA homeostasis. During cholestasis, FXR signaling mediates an adaptive response to reduce BA pool size by inhibiting BA synthesis and modulating its transport (Yuan and Li, 2016). FXR currently represents a promising target for the treatment of human cholestatic diseases (Hirschfield et al., 2015; Nevens et al., 2016; Kowdley et al., 2018). Regulation of FXR involves a dynamic deacetylation process coordinated by SIRT1 and is required for FXR-DNA binding and target gene transcription, and the same process regulates FXR proteasomal degradation (Blokker et al., 2019).

SIRT1 is an evolutionarily conserved nicotinamide adenine dinucleotide^+^-dependent histone III deacetylase which is activated in response to energy deprivation and controls key metabolic functions such as BA metabolism (Houtkooper et al., 2012; García-Rodríguez et al., 2014). Loss of hepatic SIRT1 increases BA concentrations and decreases FXR activity and expression of its target genes such as the bile salt output pump (*BSEP*) (Kemper et al., 2009; Purushotham et al., 2012). Additionally, SIRT1 inhibits hepatic BA synthesis through the small heterodimer partner (SHP)/liver receptor homolog 1 regulatory loop (Chanda et al., 2010), and it promotes hepatic FXR/RXR heterodimerization at FXRE by deacetylating FXR, primarily at Lys-217, and activates SHP and BSEP expression (Kulkarni et al., 2016). However, interactions of SIRT1 and FXR must be precisely coordinated, considering that prolonged SIRT1-mediated FXR deacetylation leads to FXR ubiquitination and proteasome degradation (Kemper et al., 2009). In line with this, hepatic SIRT1 overexpression aggravates liver injury in bile duct ligation mice (Blokker et al., 2019). Notably, administration of the SIRT antagonist SRT1720 (50 mg/day/kg body weight [mpk], orally) alleviates cholestatic liver injury in mice by increasing hydrophilicity of hepatic BA compounds and by decreasing plasma BA concentrations through increased BA excretion via urine (Kulkarni et al., 2016). These divergent findings suggest that SIRT1 exerts other regulatory mechanisms which play an important role in the development of cholestasis, which warrants further investigation.

Resveratrol, a known SIRT1 activator, can counteract ANIT-induced cholestatic liver injury mainly by regulating BA homeostasis and reducing hepatic inflammation, both of which are regulated in an FXR-dependent manner, thereby contributing to amelioration of cholestasis (Ding et al., 2018). Moreover, resveratrol effectively alleviates ANIT-induced acute cholestatic liver injury in rats, probably by increasing expression of hepatic transporter genes, thereby reducing accumulation of BAs (Wang et al., 2014). Similar to resveratrol, pterostilbene (PTE; *trans*-3,5-dimethoxy-4-hydroxystilbene), a structural analogue of resveratrol, is also an activator of SIRT1 (Liu et al., 2017). PTE occurs mainly in grapes, wine, blueberries, and other berries and has recently attracted considerable attention because of its broad pharmacological effects on various chronic human diseases (Chiou et al., 2010; Wang et al., 2015). In animals with diabetes and diet-induced obesity, PTE helps reduce body weight, plasma lipoprotein concentrations, cholesterol levels, and liver lipid accumulation (Gómez-Zorita et al., 2020; Gómez-Zorita et al., 2021). However, despite the remarkable potential of SIRT1 to modulate BA metabolism processes, the role of PTE in cholestasis has never been investigated, to the best of our knowledge. Here, we examined whether PTE treatment can alleviate cholestatic liver injury in ANIT- or DDC-induced cholestasis mouse models, and we further explored the underlying mechanisms.

## Materials and Methods

### *In vivo* Studies With Animals

All animal care and experimental protocols for *in vivo* studies conformed to the Guide for the Care and Use of Laboratory Animals published by the NIH (NIH publication no. 85–23, revised 1996). The animal studies were reported in compliance with the ARRIVE guidelines (McGrath and Lilley, 2015; Lilley et al., 2020; Percie du Sert et al., 2020). We decided sample size for animal studies based on a survey of data from published research or preliminary studies, and no mice were excluded for statistical analysis. Animal studies were approved by the Ethics Committee of the Second Clinical Medical College of Jinan University, Shenzhen People’s Hospital. Male C57BL/6J wild type mice (8 ± 0.5 weeks old) were purchased from Gempharmatech Co. Ltd (Nanjing, Jiangsu, China). These mice were maintained in SPF units of the Animal Center of Shenzhen People’s Hospital (with a 12-h light cycle from 8 a.m. to 8 p.m., 23 ± 1°C, 60–70% humidity) and maintained on a standard rodent diet with free access to water in plastic bottles. Mice were allowed to acclimatize to their housing environment for at least 7 days before experiments. Up to five mice were kept per plastic cage with corn cob bedding material. We conducted treatment to mice in a blinded fashion. The drugs used for treating animals were prepared by researchers who did not carry out the treatments. In addition, all animals were randomized before they received treatment. At the end of experiment, all mice were anesthetized and euthanized in a CO2 chamber followed by collection of liver and blood samples. According to previous study, the dose of 20 or 40 mpk PTE (oral gavage) treatment effectively improve liver fibrosis induced by high fructose feeding in rats (Song et al., 2019). Hence, in present study, the dose of 25, 50 and 100 mpk PTE (oral gavage) was selected as the concentrations *in vivo* experiment.

### ANIT-Induced Mouse Model

Male C57BL/6J wild type mice (8 ± 0.5 weeks old) were divided into six group (n = 6). And then mice were pretreated with different dose of PTE (oral gavage) or vehicle for five consecutive days. Subsequently, mice were then treated with either vehicle (corn oil) or ANIT (100 mpk; oral gavage) on day 5. All the animals were sacrificed 48 h after ANIT gavage.

### DDC-Induced Mouse Model

Male C57BL/6J wild type mice (8 ± 0.5 weeks old) were divided into six group (n = 6). C57BL/6J mice fed with a diet (control diet; Research Diets Inc, United States) containing 0.1% 3,5-diethoxycarbonyl-1,4-dihydrocollidine (DDC) for 2 weeks. After 1 week DDC treatment, mice were treated with different dose of PTE (oral gavage) for another week.

### Cell Culture

#### BMDMs

Bone marrow macrophages (BMDMS) were isolated and cultured as follows: male C57BL/6J mice (6–8 weeks of age) were sacrificed and the leg bones were separated on a sterile table. The bone cavity of femur and tibia was rinsed repeatedly with cold PBS to collect the bone marrow. The solution was then filtered with a 40 μm nylon cell filter (WHB, Shanghai, China) and centrifuged at 400 rcf for 5 min. Bone marrow granules were resuspended in DMEM containing 100 ng/mL M-CSF (Peprotech-31502) and 10% FBS (Gibco-A3160802) and 1% penicillin/streptomycin (Invitrogen-15140122) and then sow in 60 mm board, Cultured in a 37°C cell incubator (5% carbon dioxide).

### Primary Hepatocyte Isolation and Culture

Primary hepatocyte isolation was performed using a modified 2-step collagenase perfusion technique as previously reported (Xiang et al., 2021). Briefly, the liver specimen was cannulated under sterile conditions and flushed once with 50 ml washing buffer containing 2.5 mM EGTA (SIGMA-Aldrich). This was followed by perfusion with 30 ml digestion buffer containing 0.03% w/v collagenase (Roche Diagnostics) allowing recirculation of the perfusate. Removed the liver and put it in a dish of 10% FBS DMEM after infusion. The resulting cell suspension was poured through a 100 μM Cell Strainer and centrifuged with subsequent washing of the cell pellet using Serum-free medium (50 g, 2 min, 4°C). Cells were then re-suspended in William’s medium E (Biochrom AG) containing 10% fetal bovine serum. Cell number and viability were determined by the Trypan blue exclusion test. Hepatocytes were cultured using 6-well plates precoated with a single layer of rat tail collagen. Cells were seeded at a concentration of 2.5 × 10^6^ viable cells per well. Sixteen to 18 hours after plating, culture medium was changed to remove dead and non-adherent cells.

### Quantitative Real-Time Polymerase Chain Reaction, Western Blotting, and ELISA

At the end of experiment, Trizol reagent (Invitrogen) was used to extract total RNA as described in classic protocol. Chloroform was well mixed with homogenate and centrifuged at 13,300 rpm for 15 min at 4°C. The top aqueous phase was collected and mixed with isopropanol and stored at -20°C. Next day, RNA was centrifuged, and washed with 75% ethanol and 100% ethanol in sequence. Then RNA was dissolved in an appropriate amount of RNase water. The cDNA was obtained by a reverse transcription kit purchased from New England Biolab (lpswich, MA, USA). qRT–PCR was performed using the ABI StepOnePlus™ Real-time PCR system (Applied Biosystem) with specific primers (Table 1). The relative mRNA level of target genes was analyzed using equation 2–ΔCt (ΔCt = Ct of the target gene–Ct of β-actin) and normalized using the level detected in the control group as 1. After treatment, a piece of tissue or cells were lysed or homogenized in the lysis buffer (sigma-Aldrich; St. Louis, MO, United States), and total protein obtained according the classical protocol. 1:1000 diluted fresh primary antibody or anti-β-actin antibody (1:5000) in PBS containing 1% fresh dry fat-free milk, and 1:5000 diluted fresh HRP-conjugated anti-rabbit or mouse IgG in PBS containing 1% fresh dry fat-free milk. Western blotting experiments were performed using antibodies for SIRT1 (abcam; Cat:# ab110304), p53 (abcam; Cat:# ab32389), FXR (abcam; Cat:# ab187735), IκBα (abcam; Cat:# ab76429), p19Arf (abcam; Cat:# ab26696), p-IκBα (Ser32) (abcam; Cat:# ab92700), PUMA (abcam; Cat:# ab9643) and β-actin (CST; Cat:# 3700), as described previously (Yang et al., 2020). The levels of level of AST, ALT, bilirubin, and AP etc., were measured using a commercial ELISA kit (Abcam) according to the manufacturer’s protocol. The activity of SIRT1 was measured using a commercial SIRT1 Activity Assay Kit (Fluorometric) (ab156065) according to the manufacturer’s protocol.

**TABLE 1:** The sequences of primers for qRT-PCR analysis.

Gene	Forward	Backward
*mTNF-α(ID:21926)*	***GAC​GTG​GAA​CTG​GCA​GAA​GAG***	***TTG​GTG​GTT​TGT​GAG​TGT​GAG***
*mCcr2(ID:12772)*	***ATC​CAC​GGC​ATA​CTA​TCA​ACA​TC***	***CAA​GGC​TCA​CCA​TCA​TCG​TAG***
*mCcl2(ID:20296)*	***TTA​AAA​ACC​TGG​ATC​GGA​ACC​AA***	***GCA​TTA​GCT​TCA​GAT​TTA​CGG​GT***
*mCol1α1(ID:12842)*	***GCT​CCT​CTT​AGG​GGC​CAC​T***	***CCA​CGT​CTC​ACC​ATT​GGG​G***
*mCo4α1(ID:12816)*	***AAG​TTG​ACC​CAC​CTT​CCG​AC***	***GGT​CCA​CTG​TTA​TTC​TGT​AAC​CC***
*mCK-19(ID:16669)*	***GGG​GGT​TCA​GTA​CGC​ATT​GG***	***GAG​GAC​GAG​GTC​ACG​AAG​C***
*mOatp(ID:28248)*	***GGG​AAC​ATG​CTT​CGT​GGG​ATA***	***GGA​GTT​ATG​CGG​ACA​CTT​CTC***
*mNtcp(ID:20493)*	***CAA​ACC​TCA​GAA​GGA​CCA​AAC​A***	***GTA​GGA​GGA​TTA​TTC​CCG​TTG​TG***
*mAbcc2(ID:12780)*	***GTG​TGG​ATT​CCC​TTG​GGC​TTT***	***CAC​AAC​GAA​CAC​CTG​CTT​GG***
*mAbcc4(ID:239273)*	***CAT​CGC​GGT​AAC​CGT​CCT​C***	***CCG​CAG​TTT​TAC​TCC​GCA​G***
*mCyp7a1(ID:13122)*	***GGG​ATT​GCT​GTG​GTA​GTG​AGC***	***GGT​ATG​GAA​TCA​ACC​CGT​TGT​C***
*mCyp8b1(ID:13124)*	***CCT​CTG​GAC​AAG​GGT​TTT​GTG***	***GCA​CCG​TGA​AGA​CAT​CCC​C***
*mSHP(ID:19261)*	***CCA​CGG​GGA​AGG​AAC​TGA​AG***	***ACG​TAT​TCT​CCT​GCG​AAA​CTG​TA***
*mBsep(ID:27413)*	***TCT​GAC​TCA​GTG​ATT​CTT​CGC​A***	***CCC​ATA​AAC​ATC​AGC​CAG​TTG​T***
*mNos2(ID:18126)*	***GTT​CTC​AGC​CCA​ACA​ATA​CAA​GA***	***GTG​GAC​GGG​TCG​ATG​TCA​C***
*mIL-1β(ID:16176)*	***GCA​ACT​GTT​CCT​GAA​CTC​AAC​T***	***ATC​TTT​TGG​GGT​CCG​TCA​ACT***
*mIL-10(ID:16153)*	***GCT​CTT​ACT​GAC​TGG​CAT​GAG***	***CGC​AGC​TCT​AGG​AGC​ATG​TG***
*mArg1(ID:11846)*	***CTC​CAA​GCC​AAA​GTC​CTT​AGA​G***	***AGG​AGC​TGT​CAT​TAG​GGA​CAT​C***
*mRetnla(ID:57262)*	***CCA​ATC​CAG​CTA​ACT​ATC​CCT​CC***	***ACC​CAG​TAG​CAG​TCA​TCC​CA***
*mCd163(ID:93671)*	***ATG​GGT​GGA​CAC​AGA​ATG​GTT***	***CAG​GAG​CGT​TAG​TGA​CAG​CAG***
*mFxr(ID:20186)*	***GCT​TGA​TGT​GCT​ACA​AAA​GCT​G***	***CGT​GGT​GAT​GGT​TGA​ATG​TCC***

#### Flow Cytometry

Mice were anesthetized with 3% sodium pentobarbital, rapidly exposed to the liver and infused with 20 ml cold phosphate buffer saline (Python (Hyclone-C11995500BT) containing 50 U/mL hyaluronidase (Sigma-Aldrich-H3506). 60 U/mL DNASE1 (Invitrogen-18047019), and 450 U/mL Type I collagenase (Sigma-Aldrich-C0130), for 45 min at 37°C on a shaker. After digestion, the solution was votexed for 20 s and then passed through a 40 μm cellular filter (WHB-40) to add 10 ml HBSS containing 2% FBS and 0.2% BSA. Cells were centrifuged at 400 rcf for 5 min (4°C) and resuspend with 1 ml Stain buffer (FBS). After the second centrifugation (400 rcf, 5 min, 4°C), the cells were resuspend in 100 μl staining buffer (FBS) containing 1% anti-CD16/CD32 (BD-553141). After incubation at room temperature for 15 min, the cells were stained and incubated at 4°C in dark for 30 min. The antibody of BD Biosciences: anti-CD45 APC-CY7 (BD-557659 1:100), Anti-CD11b FITC (BD-557396, 1:100), Anti-F4/80 PE (BD-565410, 1:100) was added. Cells were directly classified into PBS on the FACS ARIA II cell classifier (BD Biosciences) for subsequent RNA isolation. After the incubation, the mixture was centrifuged at 400 rcf for 5 min, the supernatant was discarded, and 400ul Stain buffer was added to resuspend.

#### Histological Analysis

The mice tissues were preserved with 4% paraformaldehyde solution, dehydrated and embedded in paraffin. Sections (4 μm) were used for histological analysis. HE, Sirus red, immunofluorescence [α-SMA (CST; Cat:# 19245), CD80 (abcam; Cat:# ab225674), F4/80 (abcam; Cat:# ab6640)]**,** and TUNEL staining (abcam; Cat:# ab66108) performed as standard instructions. Positive cells were morphometrically quantified by a technician (blinded to the treatments) with image processing software (ImageJ). The TUNEL-positive cells were counted in the liver in three fields per animal at×100 magnification, and the number of TUNEL-positive neurons per millimeter squared in ANIT group as control 1.

#### Molecular Docking

Discovery Studio (DS) 2019 is a molecular modeling software for protein structure studies and drug discovery (Zhang et al., 2020). The 2D structure of small molecule drug and the crystal structure of target protein were downloaded from PubChem (https://www.ncbi.nlm.nih.gov/pccompound) and PDB database (https://www.rcsb.org), respectively. Small molecule drugs and target proteins were used as ligands and receptors respectively in molecular docking by DS software. First, the small molecule drug was used for ligand preparation, a method to remove duplicates, enumerating isomers and tautomers, and generating 3D conformations. Next, a series of preparations were also applied to the protein receptor, including removing water molecules, adding hydrogen atoms, setting up active pockets, etc. Finally, CDocker was used for molecular docking, an algorithm that allows precise docking of any number of ligands to a single protein receptor (Wu et al., 2003). -Cdocker Interaction Energy was used to evaluate the binding ability between small molecule drugs and target proteins, which was compared with the ligands and receptors that have been clearly bound.

### Analysis of p53 Ubiquitination

Immunoprecipitation of ubiquitinylated proteins was performed using the UbiQapture-Q Kit (Enzo Life Sciences), following the manufacturer’s instructions. Briefly, protein samples were harvested from BMDM and protein concentration was measured by BCA assay. Ubiquitinylated proteins were captured from 25 μg of total proteins lysates using 40 μl UbiQapture-Q matrix. Captured and uncaptured fraction samples were analyzed by Western blotting, as detailed above.

#### Immunoprecipitation

To determine acetylation status of Fxr in liver homogenates, 1-mg whole-liver extracts were incubated with antibody to FXR overnight under stringent conditions, immunopurified using Dynabeads Protein G beads (Life Technologies Corporation), and immunoblotted using Ac-Lysine (Cell Signaling Technology).

#### Data Analysis

Based on our previous studies and/or preliminary experiments, we calculated the group size for *in vitro* studies. No outliers were identified in the reported experiments, and no data were excluded from analysis. All the data were generated from at least five independent experiments. The density of target band and qRT-PCR target gene mRNA was normalized to β-actin in the corresponding sample to reduce variance. All values (control and test) were normalized to the mean value of the experimental control group. The data were expressed as % of the control group’s mean value. After captured, the density of image was quantified by person who was blinded to the treatment with ImageJ software (National Institutes of Health, Bethesda, MD, United States). All the raw data were initially subjected to a normal distribution anal analysis with SPSS 22 software (1-sample K-S of nonparametric test). For ANOVA, Tukey’s post hoc test was performed for data with F at *p* < 0.05 and no significant variance inhomogeneity. The declared group size is the number of independent values, and that statistical analysis was done using these independent values. Correlation coefficient was calculated using Spearman correlation test and one-tailed *p*-value was calculated for 95% confidence interval. The significant difference was considered at *p* < 0.05 (n ≥ 5).

## Results

### Effects of PTE on ANIT-Induced Cholestasis

**FIGURE 1:**
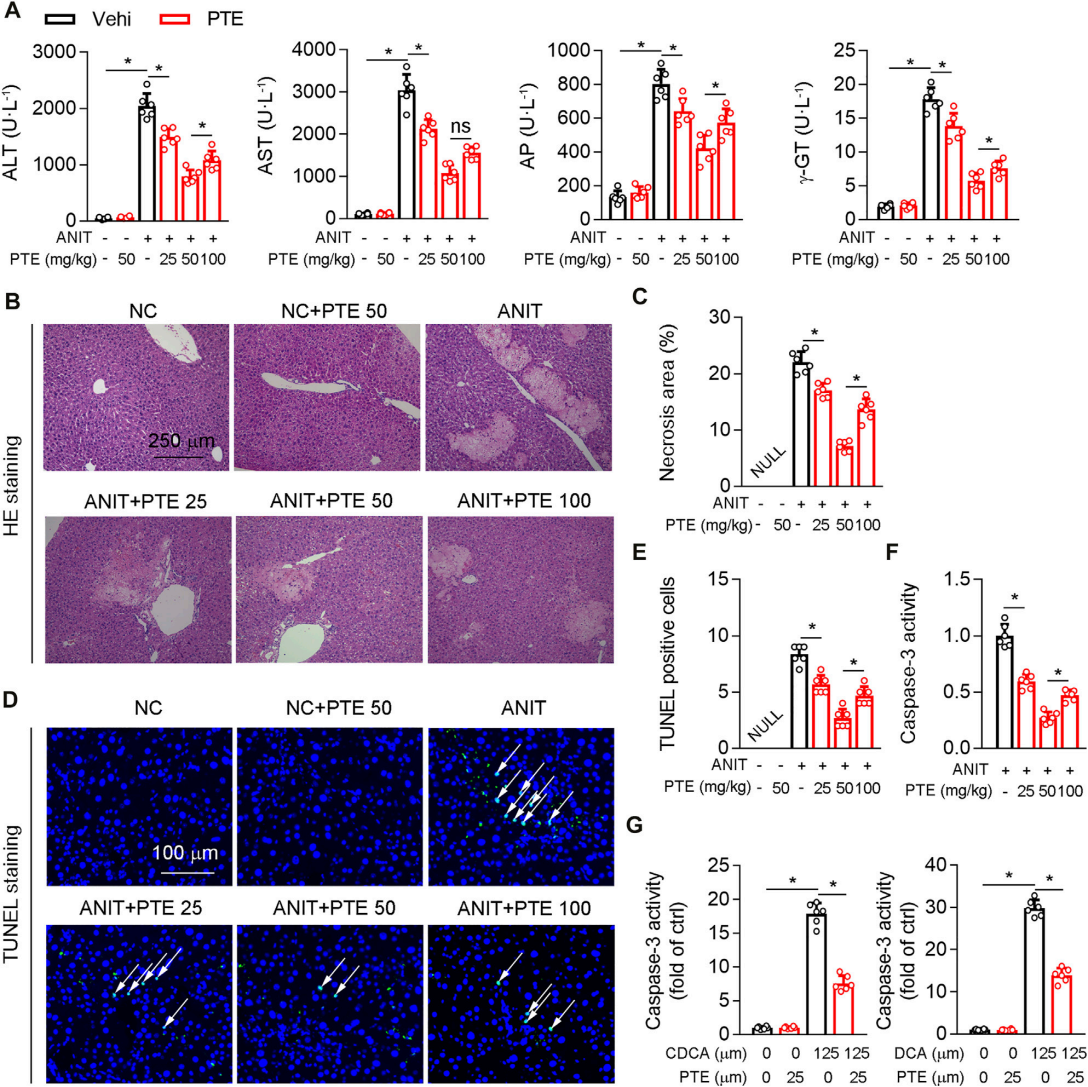
PTE improved liver injury in an ANIT-induced mouse model of cholestasis. C57BL/6J mice intragastrically administered with 25, 50 or 100 mpk PTE daily for continuous 5 days. On the fifth day, the mice were also gavaged with ANIT (100 mpk) in addition to PTE. The mice were sacrificed 48 h after ANIT intragastric administration **(A)** Serum ALT, AST, AP, and γ-GT was determined by Elisa kit, n = 6 **(B,C)** Representative images of hepatic HE staining were shown and quantification of apoptosis areas were shown in the right panel, n = 6 (the results quantification were determined in the liver in three fields per animal at×100 magnification) **(D,E)** Representative images of TUNEL staining and quantification of positive cells were shown, n = 6 (the TUNEL-positive cells were counted in the liver in 1 fields per animal at×100 magnification, and the number of TUNEL-positive neurons per millimeter squared in ANIT group as control 1) **(F)** Activity of caspase-3 in mouse liver was determined by following the kit instructions, n = 6 **(G)** Caspase-3 activity was determined in primary hepatocytes isolated from WT mice in the presence of chenodeoxycholic acid (CDCA) and DCA with or without PTE. *p < 0.05. The data represent the mean ± SD.

Treatment of mice with ANIT or feeding 0.1% DDC are commonly accepted methods of establishing sclerosing cholangitis which is similar to PSC (Fickert et al., 2007; Fickert et al., 2014); we thus assessed hepatoprotective effects of PTE using such ANIT or DDC-induced CLD models. Various dosages of PTE alleviated ANIT-induced liver injury, as evidenced by reduced levels of serum aspartate transaminase (AST), alanine transaminase (ALT), alkaline phosphatase (AP), and γ-GT (Figure 1A). Moreover, PTE intervention reduced the size and number of ANIT-induced necrosis (assessed using HE staining) and apoptotic cell death (assessed using TUNEL staining) (Figures 1B–E). ANIT treatment increased caspase-3 activity in mouse livers, which was reduced after PTE treatment (Figure 1F). Compared with the 25 or 100 mpk PTE tratment, 50 mpk ameliorated ANIT-induced liver injury more effectively. *In vitro* analyses of primary hepatocytes isolated from WT mice confirmed that PTE treatment desensitized liver cells to bile-acid–induced apoptotic cell death (Figure 1G).

### PTE Ameliorated DDC-Induced Experimental Cholestasis

**FIGURE 2:**
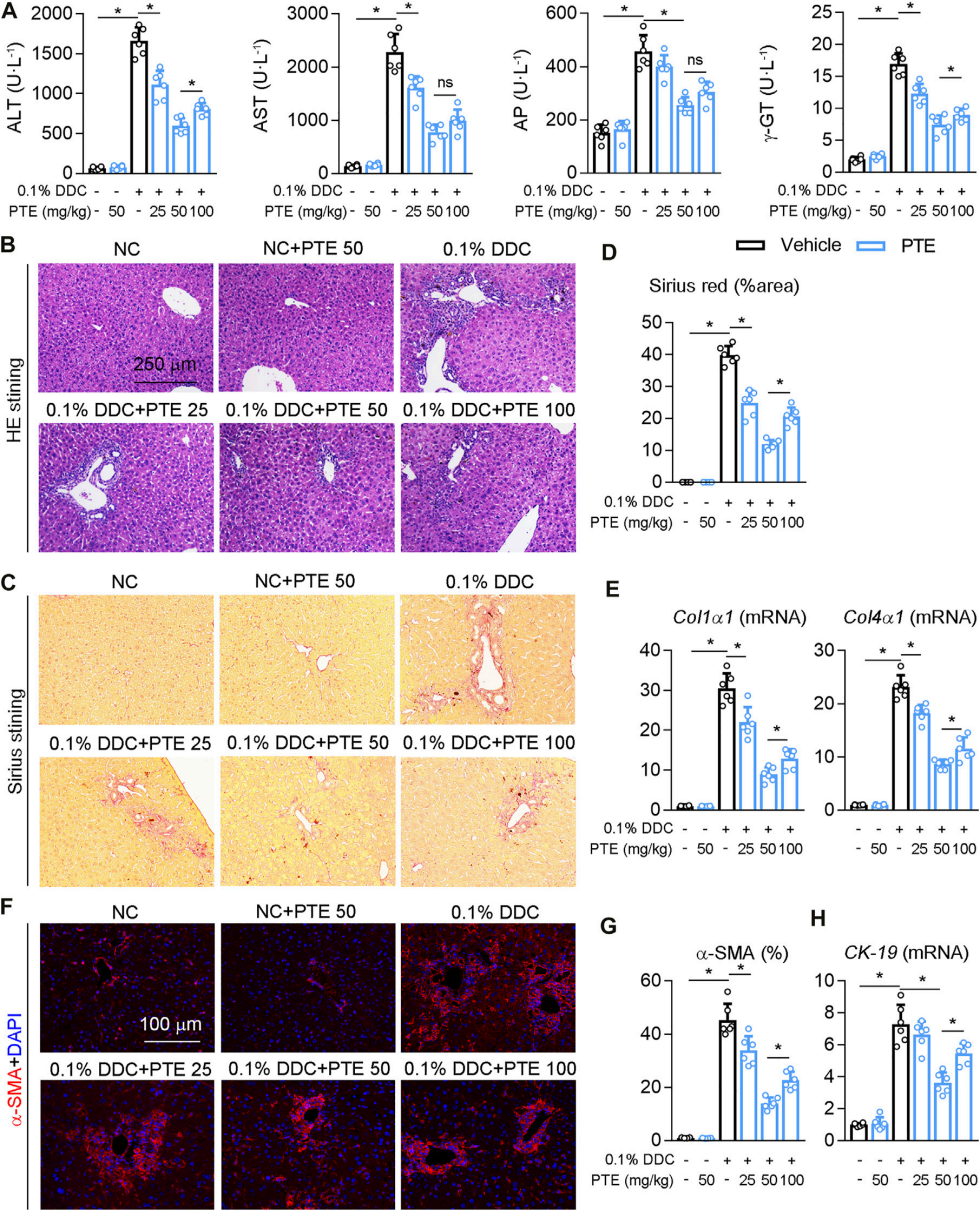
PTE intervention reduced DDC-induced liver injury in mouse model of cholestasis. C57BL/6J mice were fed with 0.1% DDC for 2 weeks (C57BL/6J mice were fed with normal chow food as control group). After 1 week, mice intragastrically administered with 25, 50 or 100 mpk PTE daily for 1 week **(A)** Serum ALT, AST, AP, and γ-GT was determined by Elisa kit, n = 6 **(B**–**D)** Representative images of hepatic HE staining **(B)** and Sirius red staining **(C)** were shown, and quantification of Sirius red were shown in the panel D, n = 6 (the results quantification were determined in the liver in 1 fields per animal at×100 magnification) **(E)** qPCR analysis of *Col1α1* and *Col4α1* in mouse liver, n = 6 **(F,G)** Representative immunofluorescence results of α-SMA were shown in the panel F, and the quantitative results are shown in the panel G, n = 6 **(H)** qPCR analysis of *CK-19* in mouse liver, n = 6. *p < 0.05. The data represent the mean ± SD.

To further confirm the role of PTE in the experimental CLD, C57BL/6J mice fed with PTE in a diet containing 0.1% DDC for 2 weeks. PTE treatments at 25, 50, and 100 mpk alleviated DDC-induced cholestasis in C7BL/6J mice, as shown by decreasing cholestasis-serum markers (AST, ALT, AP, and γ-GT) (Figure 2A). Consistent with this, the pathological analysis of liver was also supported our finding, PTE treatment improved liver damage induced by DDC fed (Figure 2B). In addition, hepatic fibrosis (assessed using Sirius red stain), α-SMA positive cells, and mRNA levels of *Col1α1*, *Col4α1,* and *CK-19*, compared with DDC-fed controls (Figures 2C–H). The 50 mpk PET treatment ameliorated DDC-induced liver damage more effectively than the 25 or 100 mpk treatments. Taken together, PTE (50 mpk) treatment attenuated liver damage caused by ANIT or DDC.

### Effects of PTE on Hepatic Inflammation in DDC- or ANIT-Induced Cholestasis

**FIGURE 3:**
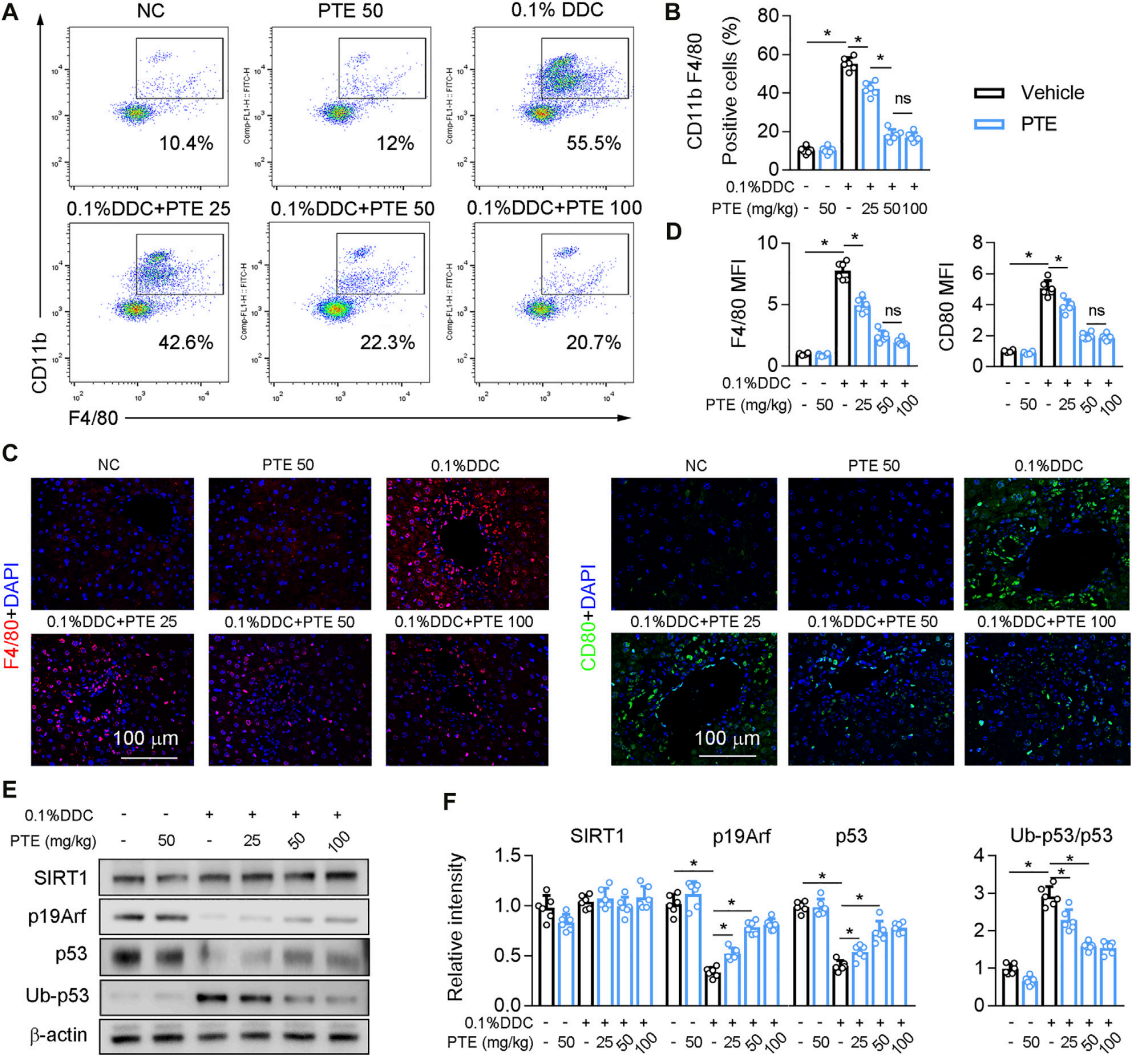
PTE inhibited DDC-induced hepatic macrophage infiltration and activation through SIRT1-p19-p53 signal pathway **(A,B)** liver-isolated immune cells were stained for CD11b and F4/80 and analyzed by flow cytometry, representative dot plots are shown (A). The quantification of flow cytometry were shown in panel B, n = 6 **(C,D)** Representative immunofluorescence results of F4/80 and CD80 (Macrophage activation marker) were shown in the panel C, and the quantitative results were shown in the panel D, n = 6 **(E,F)** Western blot analysis was used to confirm the expression of SIRT1, p19Arf, p53, and Ub-p53 **(E)**, and quantification of results were shown in the panel F, n = 6. *p < 0.05. The data represent the mean ± SD.

The recruitment of monoctyes and macrophages is significantly increased in the liver of patients with cholestasis (Ma and Chen, 20191979). Macrophage recruitment to the liver was decreased in PTE-treated CLD model mice, compared with mice treated with DDC only (Figures 3A,B), and it was significantly lower in the 50 mpk than in the 25 mpk PTE treatment (Figures 3A,B). Hepatic macrophage recruitment did not differ significantly between the 50 and 100 mpk PTE treatments (Figures 3A,B). F4/80 immunofluorescence staining produced comparable results (Figures 3C,D). Furthermore, PTE treatment inhibited macrophage activation (M1 macrophage polarization) in the liver of DDC-induced CLD mouse models, as evidenced by fewer CD80-positive cells in the liver after PTE treatment (Figures 3C,D). Liver macrophage activation was significantly reduced in the 50 and 100 mpk treatments, compared to the 25 mpk treatment; however, no significant difference was observed between the 50 and 100 mpk treatments. Previous studies highlight an anti-inflammatory function of p53 in a variety of pathophysiological conditions (Zheng et al., 2005; Liu et al., 20091950). Evidence suggests that SIRT1 activation increased p19, which inactivates MDM2 E3 ligase and sustains p53 activity (Alarcon-Vargas and Ronai, 2002; Nakamura et al., 2017). Western blotting of SIRT1, p19Arf, p53, and ubiquitinylated-p53 (Ub-p53) of protein levels showed that SIRT1 expression was slightly but not significantly decreased in the liver of mice treated with DDC, compared with those of controls, and DDC treatment significantly inhibited p53 and p19Arf expression but increased Ub-p53 (Figures 3E,F). Compared with mice treated with DDC only, PTE treatment at different dosages had no significant influence on SIRT1 expression but increased p19Arf and p53 expression and decreased Ub-p53. DDC treatment induced expression of macrophage activation markers (*IL-1β, TNF-α, Ccr2,* and *Nos2*) and reduced macrophage alternative activation (Supplementary Figure S1A). PTE treatment significantly inhibited macrophage activation gene expression and slightly increased expression of macrophage alternative activation genes. Similar changes were observed in the ANIT-induced CLD models (Supplementary Figure S1B). Taken together, PTE inhibited hepatic inflammation in DDC and ANIT-induced experimental intrahepatic cholestasis.

### Effects of PTE on Bile Metabolic Dysfunction in DDC- or ANIT-Induced Cholestasis

**FIGURE 4:**
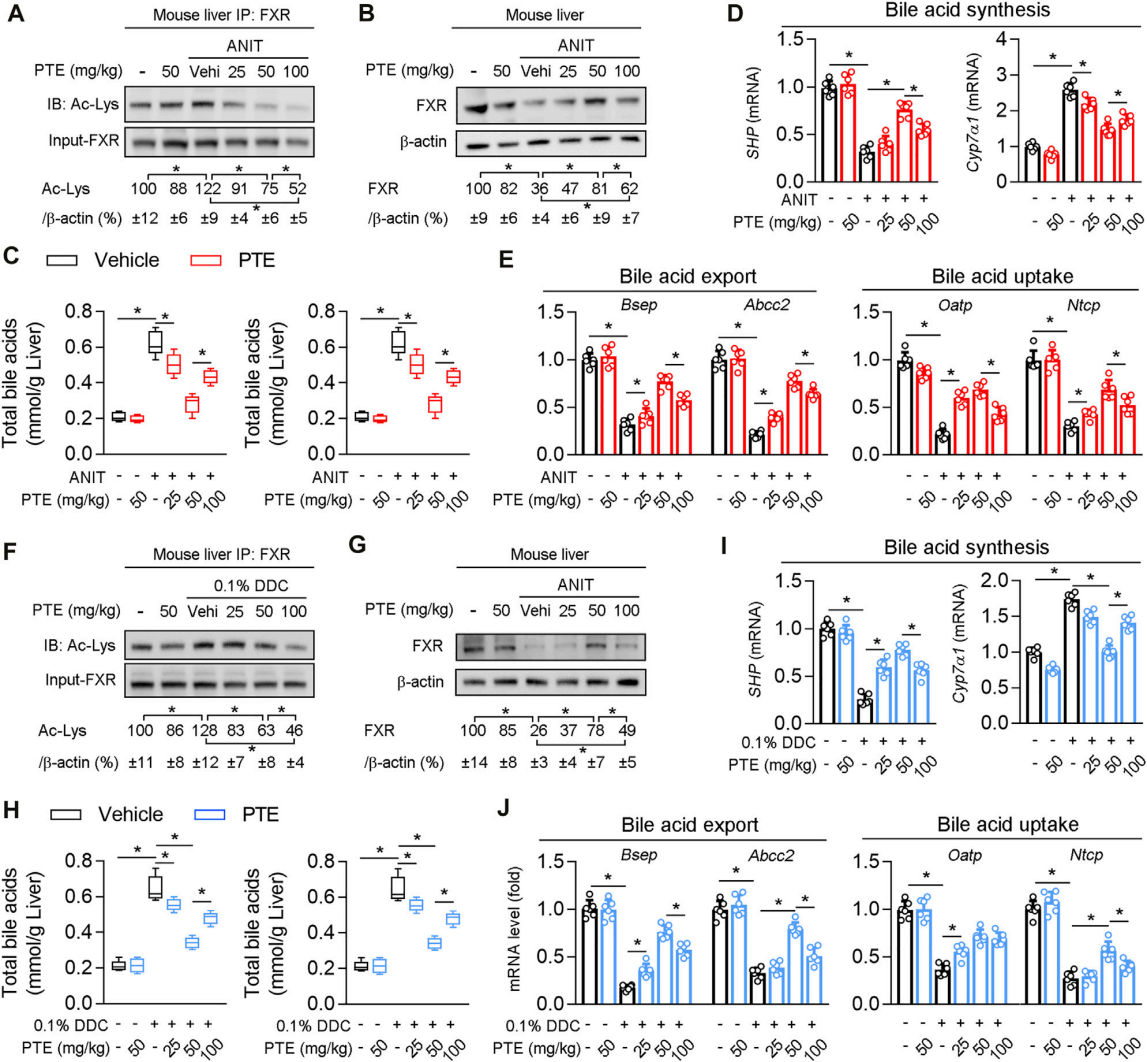
PTE treatment correlated with higher presence and activity of FXR and lower accumulation of bile acids in liver of CLD mouse **(A)** Liver whole cell lysates from ANIT-induced mice were immunoprecipated with FXR antibody and immunoblotted using Ac-Lys antibody, n = 6 **(B)** Western blot analysis was used to confirm the expression of FXR and β-actin in whole liver of mice, n = 6 **(C)** Quantification of bile acid pool size in livers by Elisa kit, n = 6 **(D,E)** qPCR analysis of *SHP*, *Cyp7α1* and bile acid transporters in mouse liver, n = 6 **(F)** Liver whole cell lysates from DDC fed mice were immunoprecipated with FXR antibody and immunoblotted using Ac-Lys antibody, and quantification results were shown in the right panel, n = 6 **(G)** Western blot analysis was used to confirm the expression of FXR and β-actin in whole liver of mice, n = 6 **(H)** Quantification of bile acid pool size in livers by Elisa kit, n = 6 **(I,J)** qPCR analysis of *SHP*, *Cyp7α1* and bile acid transporters in mouse liver, n = 6. *p < 0.05. The data represent the mean ± SD.

Western blotting of FXR protein levels in the liver showed that protein levels of FXR were significantly decreased and acetylation of FXR slightly but not significantly increased in ANIT-treated mice, compared to the controls (Figures 4A,B). ANIT-induced CLD model mice treated with 25 mpk PTE showed only slightly upregulated FXR expression, whereas the 50 and 100 mpk treatments produced significantly increased FXR expression; however, FXR expression was lower in the 100 mpk PTE treatment than in the 50 mpk treatment. Liver BA pool sizes were increased in mice treated with ANIT only, compared to the controls, whereas a significant decrease was observed in PTE-treated mice (Figure 4C). Lower expression of *SHP*, *Bsep*, ATP binding cassette subfamily C member 2 (*Abcc2*), organic anion transporting polypeptide (*Oatp*), and sodium taurocholate cotransporting polypeptide (*Ntcp*) and higher cholesterol seven hydroxylase (*Cyp7A1*) in mice treated with ANIT only but not with PTE confirmed impaired FXR signaling (Figures 4D,E). PTE treatment attenuated ANIT-induced effects on BA synthesis genes and transporters; however, 100 mpk PTE adversely affected the FXR signaling pathway, compared with the 50 mpk treatment. Similar changes were observed in DDC-induced CLD models (Figures 4F–J). Overall, our results demonstrate that PTE treatment improved bile metabolic dysfunction induced by ANIT or DDC.

### PTE Regulated Bile Metabolic Related Gene Expression via SIRT1/FXR Signaling Pathway

**FIGURE 5:**
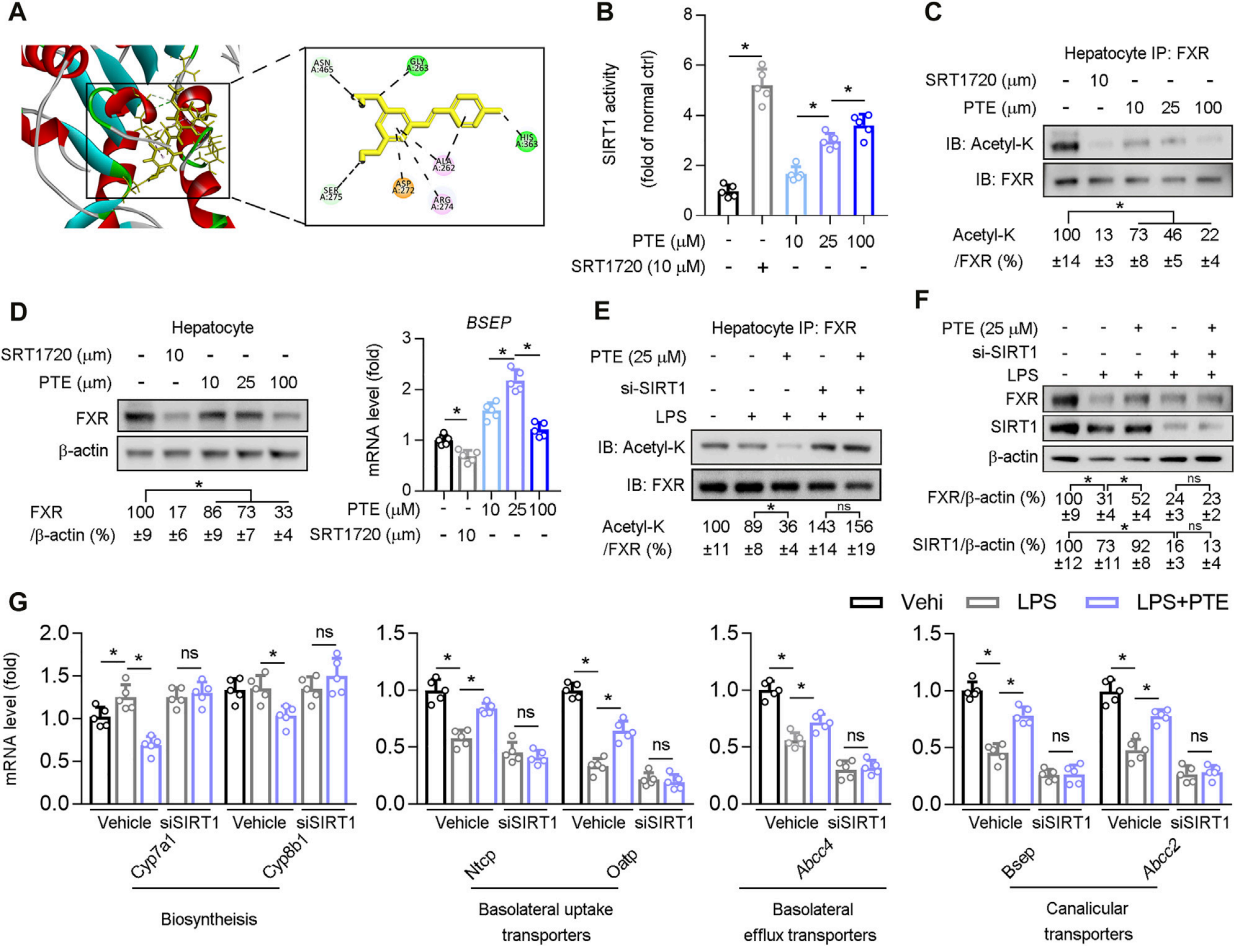
PTE regulated bile metabolic related gene expression via SIRT1/FXR signaling pathway **(A)** A representation of the computationally predicted cluster of PTE bound to SIRT1 **(B**–**C)** Primary hepatocytes isolated from WT mice treated with PTE or SRT1720 (SIRT1 agonists). B: The activity of SIRT1 determined by SIRT1 Activity Assay Kit, n = 5. C: Whole cell lysates from primary hepatocytes were immunoprecipated with FXR antibody and immunoblotted using Ac-Lys antibody, n = 5 **(D)** Western blot analysis was used to confirm the expression of FXR and β-actin in primary hepatocyte cells, and qPCR analysis of *Bsep* were shown in the right of panel D, n = 5 **(E**–**G)** Primary hepatocytes cells treated with LPS, si-SIRT1, and PTE shown in the panel E. E: Whole cell lysates from primary hepatocytes cells were immunoprecipated with FXR antibody and immunoblotted using Ac-Lys antibody. F: western blot analysis was used to determine protein level of FXR, SIRT1, and β-actin in primary hepatocytes cells, the quantitative results were shown in the right panel, n = 5. G: qPCR analysis of *Cyp7a1, Cyp8b1, Ntcp, Oatp, Abcc4, Bsep,* and *Abcc2* in primary hepatocytes isolated from WT mice, n = 5. *p < 0.05. The data represent the mean ± SD. Abbreviations: IB, immunoblotting; IP, immunoprecipitation.

Structural modeling of the PTE-SIRT1 interaction was performed using BIOVIA Discovery Studio (Figure 5A), and key interface residues in SIRT1 are shown in the stick representation and are labeled according to residue names and positions. *In vitro* protein kinase assays demonstrated that PTE activated biochemical activity of SIRT1 in primary hepatocytes isolated from wild type mice (Figure 5B). PTE treatment reduced FXR acetylation and protein levels in a dose-dependent manner (Figures 5C,D). Furthermore, mRNA of *Bsep* (an FXR regulated gene) was significantly increased at 25 μm PTE compared to that at 10 and 100 μm PTE (Figure 5D). To test whether PTE regulated FXR acetylation and activity through SIRT1, primary hepatocytes were treated with PTE and/or siSIRT1 in presence of LPS (Figures 5E,F), showing that PTE did not affect FXR acetylation and protein levels in absence of SIRT1. Furthermore, when SIRT1 was deficient, PTE treatment did not affect expression of *Cyp7a1, Cyp8b1, Ntcp, Oatp, Abcc4, Bsep*, and *Abcc2* (Figure 5G). Taken together, PTE regulated bile metabolic genes through SIRT1-FXR signaling.

### Effects of PTE on Macrophage Activation Through SIRT1 and p53 Ubiquitination

**FIGURE 6:**
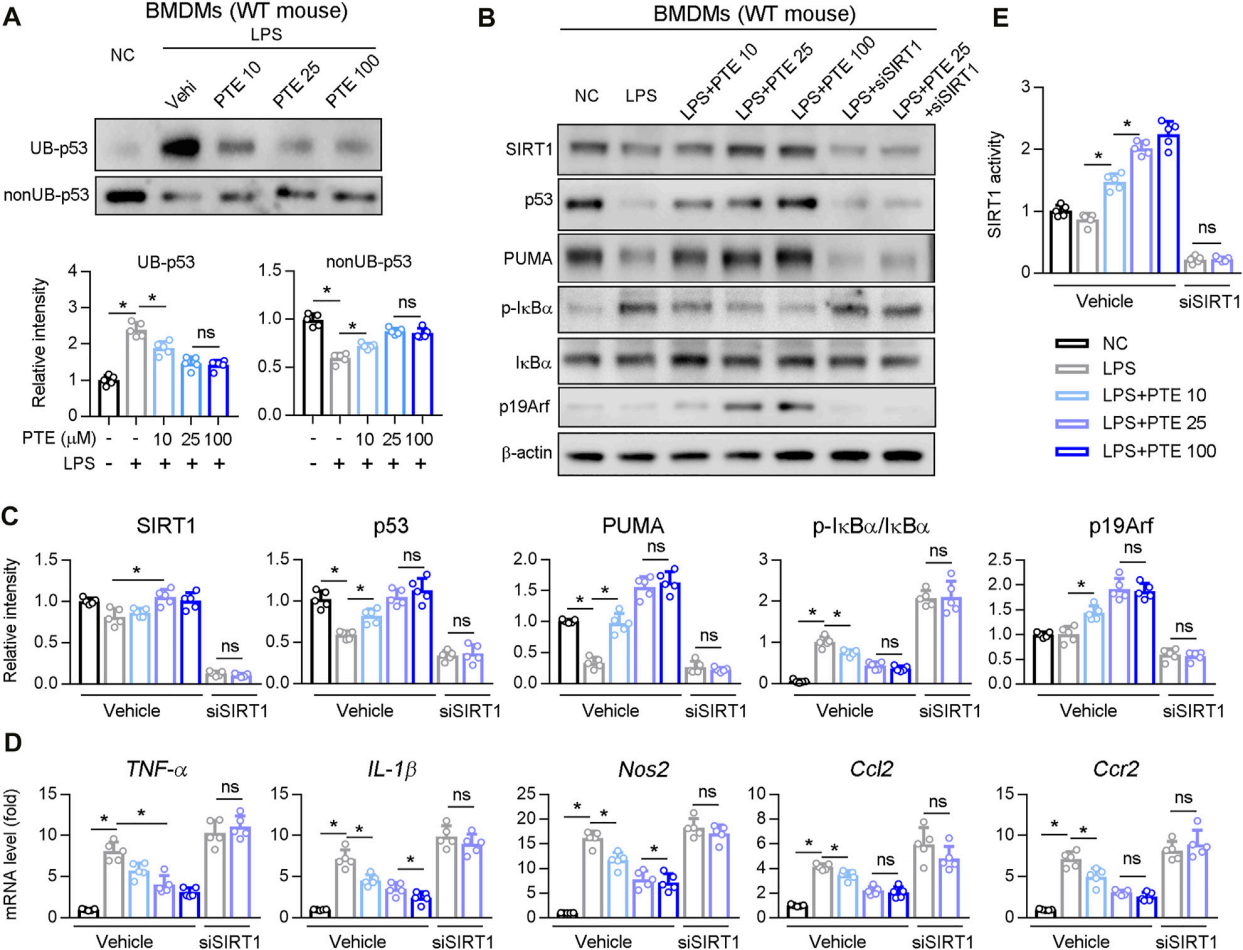
PTE inhibited macrophage activation induced by LPS through SIRT1-p53 signaling pathway **(A)** BMDMs treated with PTE and/or LPS as shown in the panel A. The ubiquitination and non-ubiquitination level of p53 was determined as described in Methods, and quantitative results were shown in the bottom panel, n = 5 **(B**–**E)** BMDMs treated with PTE, LPS or siSIRT1 as shown in the panel B. B: western blot analysis was used to detect the protein level of SIRT1, p53, PUMA, IκBα, p19Arf, and the phosphorylation of IκBα. C: the quantitative results of panel B, n = 5. D: qPCR analysis of macrophage activation marker, n = 5. E: The activity of SIRT1 determined by SIRT1 Activity Assay Kit, n = 5. *p < 0.05. The data represent the mean ± SD. Abbreviations: UB, ubiquitination.

Next, we investigated whether PTE regulated p53 ubiquitination in BMDM cultures. PTE inhibited LPS-induced p53 ubiquitination in BMDMs (Figure 6A), whereas no significant difference occurred between the 25 and 100 μm treatment groups. Western blotting of LPS-stimulated BMDM cultures supplemented with PTE or siRNA-SIRT1 showed that PTE treatment increased expression of p19Arf, p53 and p53 downstream effector protein PUMA, and reduced macrophage activation markers (i.e., phosphorylation of IκBα and mRNA levels of *TNF-α*, *IL-1β*, *Nos2*, *Ccl2*, and *Ccr2*); this pattern that was reversed after SIRT1 silencing (Figures 6B–D). In addition, PTE increased SIRT1 activation in BMDMs, and was significantly inhibited in the si-SIRT1 treatment (Figure 6E).

### Comparison of UDCA and PTE in Experimental Cholestasis

**FIGURE 7:**
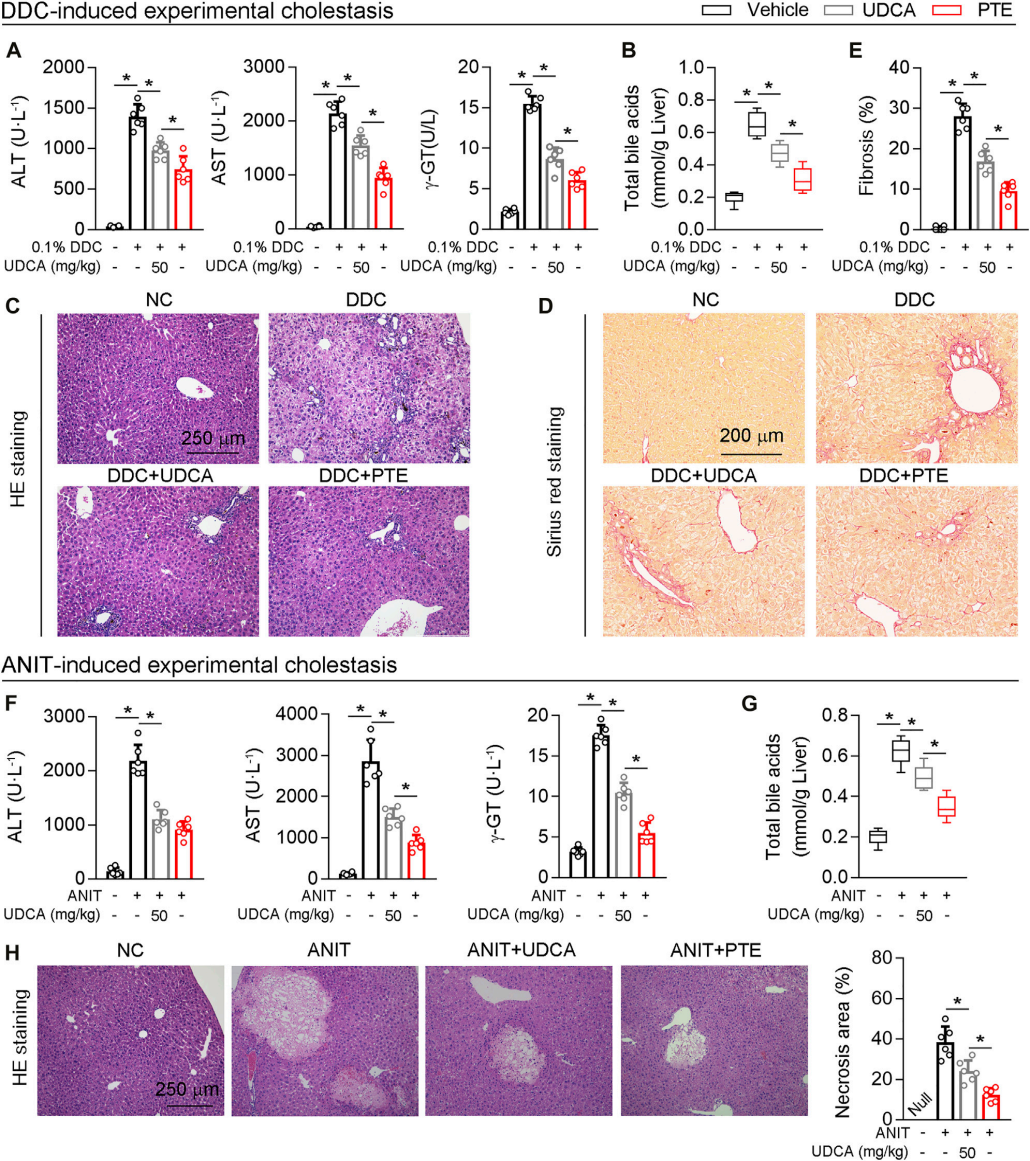
Compared with UDCA, PTE at the same dose was more effective in improving cholestatic liver injury caused by DDC or ANIT **(A)** The serum level of AST and ALT determined by Elisa kit, n = 6 **(B)** Quantification of bile acid pool size in livers by Elisa kit, n = 6 **(C**–**E)** Representative images of hepatic HE staining **(C)** and Sirius red staining **(D)**, the quantitative results of panel D were shown in the panel E, n = 6 **(F)** The serum level of AST and ALT determined by Elisa kit, n = 6 **(G)** Quantification of bile acid pool size in livers by Elisa kit, n = 6 **(H)** Representative images of hepatic HE staining and the quantitative results were shown in the right panel, n = 6. *p < 0.05. The data represent the mean ± SD.

Next, UDCA was used as positive control, and the efficacy of PTE on cholestasis induced by DDC or ANIT was compared with UDCA. According to our results, both UDCA (50 mpk) or PTE (50 mpk) treatment alleviated DDC-induced liver injury, as seen by downregulation of serum AST, ALT, γ-GT, the accumulation of TBA, and hepatic fibrosis (assessed using Sirius red stain) (Figures 7A–E). Additionally, compared with the 50 mpk UDCA tratment, 50 mpk PTE could furhter ameliorate DDC-induced liver injury (Figures 7A–E). The similar results were observed in ANIT-induced experimental cholestasis (Figures 7F–H).

## Discussion

We found that PTE treatment attenuated ANIT- or DDC-induced cholestasis. Mechanistic analyses showed that PTE inhibited macrophage infiltration and activation in the mouse liver through the SIRT1-p53 signaling pathway, and it improved hepatic bile metabolism through the SIRT1-FXR signaling pathway.

Interestingly, our results regarding regulation of FXR expression by PTE differed between *in vivo* and *in vitro* experiments. In primary hepatocytes, PTE treatment dosage was positively correlated with FXR deacetylation and was negatively correlated with FXR protein levels (Figure 5C). However, in the liver of ANIT-induced CLD mouse models, FXR was increased by approximately 2-, 3.82-, and 2.41-fold after treatment with 25, 50, and 100 mpk PTE, respectively (Figure 4B). Previous studies found that macrophage-derived inflammatory factors (IL-1β, among others) activated NF-kB-p65 in hepatocytes, resulting in binding of NF-kB-p65 to the FXR promoter region, which precluded expression of FXR (El Kasmi et al., 2018; Xiang et al., 2021). Consistent with this, when SIRT1 was absent from BMDMs, no significant change in expression of inflammatory factors (*Ccl2*, *TNF-α*, *Ccr2*, and *IL-1β*) was observed after PTE treatment of DDC-induced CLD model mice (Figure 7G); however, FXR protein levels in the liver were decreased (Figure 7I). Considering this finding, PTE regulated FXR activity in the liver of CLD mice through two distinct mechanisms: 1) PTE inhibited macrophage infiltration and activation as well as its-derived inflammatory factor (Figure 3A, Figure 3B, Figure 3C, and Supplementary Figure S1A), resulting in high level of FXR; 2) PTE enhanced SIRT1-mediated deacetylation of FXR (Figure 5C), thereby increasing its DNA binding and associated gene transcription, but SIRT1-mediated FXR deacetylation also led to ubiquitination and proteasomal degradation. Importantly, hepatic inflammation was comparable between the 50 mpk and 100 mpk PTE treatments (Figure 3F); however, FXR deacetylation was higher in the 100 mpk than in the 50 mpk treatment, resulting in increased FXR degradation (Figure 4A and Figure 4E).

SIRT1 expression can influence p53 function through two distinct mechanisms which have opposed effects on net p53 activity: 1) SIRT1 deacetylates and promotes p53 ubiquitin-related degradation (Zheng et al., 2005; Liu et al., 20091950), 2) and may also upregulate p19Arf to inhibit MDM2 E3 ligase-mediated p53 ubiquitin-related degradation (Chua et al., 2005). Thus, the effect of SIRT1 on cellular p53 activity may depend on cell types and on the context of cellular stress (Chua et al., 2005). Here, we found that PTE reduced BMDMs activation and infiltration in the liver of CLD model mice (Figures 3A–H). Moreover, PTE inhibited LPS-mediated M1 polarization in BMDMs *in vitro* (Figure 6D). Mechanistic analysis showed that PTE increased p53 expression by downregulating p53 ubiquitination (Figure 6A). In line with this, the PTE treatment significantly increased expression of p19Arf, which may inhibit MDM2-dependent ubiquitination of p53 and sustain p53 activity (Figure 6B) (Alarcon-Vargas and Ronai, 2002; Nakamura et al., 2017). An increase in the p53 downstream effector protein PUMA after PTE treatment also supported our finding that PTE enhanced p53 activity (Figure 6B). In addition, SIRT1 siRNA silencing results suggested that PTE-inhibited macrophage activation in BMDMs was SIRT1-dependent (Figures 6B–D).

Obeticholic acid (OCA), a synthetic bile acid derivative, is the first drug approved by the FDA and EMA for the treatment of PBC patients with an inadequate response to therapy with UDCA (Nevens et al., 2016; Kowdley et al., 2018). OCA is a potent agonist of the nuclear bile acid receptor farnesoid X receptor (FXR) (Lew et al., 2004). In current study, we have proven that PTE is a potential agonist of FXR both *in vivo* and *in vitro*. In addition, combination therapy with UDCA and OCA provided satisfactory clinical outcomes, which may be a promising alternative for patients with PBC who had an inadequate response to UDCA therapy (Huang et al., 2020). Hence, it is worth to detect whether PTE is superior to OCA, or combination of PTE + UDCA is superior to each one of them alone in subsequent studies.

**FIGURE 8:**
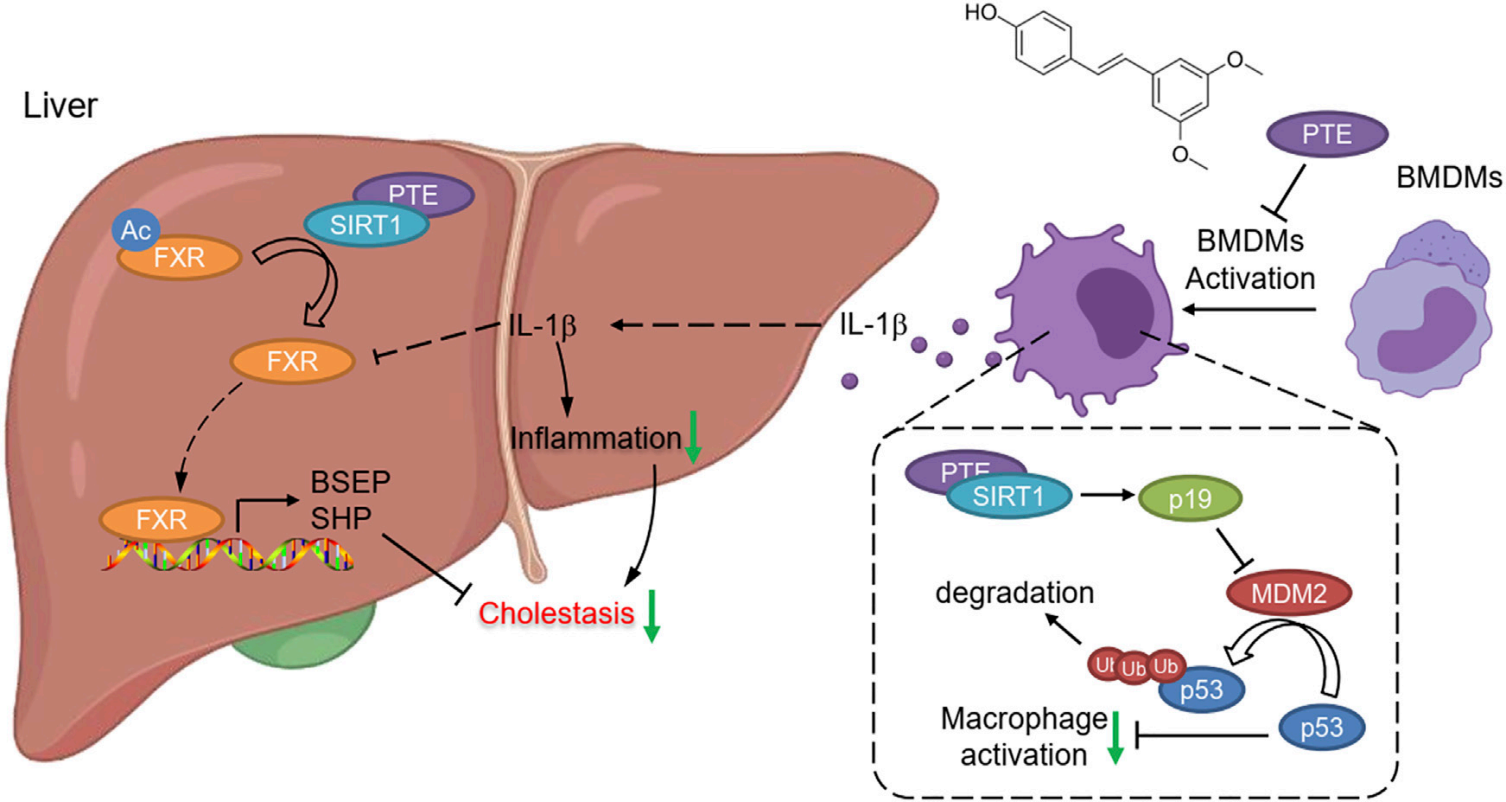
Graphical Abstract. PTE enhanced SIRT1 activity, 1) SIRT1 deacetylates FXR, enhancing its DNA binding and dependent gene transcription; 2) SIRT1 increased p19Arf activation, resulting in inhibited MDM2 activity as well as MDM2 mediated ubiquitin-related degradation of p53.

With evidences from mouse CLD samples, we have identified PTE as a novel clinical candidate compound for CLD. Collectively, our findings support the following model of mechanistic integration between PTE, SIRT1, FXR and p53 signaling (Figure 8). PTE enhanced SIRT1 activity, 1) SIRT1 deacetylates FXR, enhancing its DNA binding and dependent gene transcription; 2) SIRT1 inhibited the ubiquitin-related degradation of p53, resulting in inhibited the reduction of FXR expression mediated by the inflammatory factors-derived from M1 macrophage.

## Data Availability

The original contributions presented in the study are included in the article/Supplementary Material, further inquiries can be directed to the corresponding authors.

## Abbreviations

ALT, alanine transaminase; ANIT, α-naphthylisothiocyanate; AP, alkaline phosphatase; AST, aspartate transaminase; ATP, adenosine triphosphate; BDL, bile duct ligation; BMDMs, bone marrow-derived macrophages; BSEP, bile salt export pump; CCR-2, C–C chemokine receptor type-2; CLD, cholestatic liver disease; DDC, 3,5-diethoxycarbonyl-1,4-dihydrocollidine; FXR, farnesoid X receptor; IL-1β, interleukin 1 beta; LPS, lipopolysaccharide; OCA, obeticholic acid; PBC, primary biliary cholangitis; PSC, primary sclerosing cholangitis; TBA, total bile acids; UDCA, ursodeoxycholic acid.
