# Supplementary material for: Pterostilbene Alleviates Cholestasis by Promoting SIRT1 Activity in Hepatocytes and Macrophages
Source: Front Pharmacol. 2021 Nov 25;12:785403. doi: 10.3389/fphar.2021.785403 (PMC8656168; doi:10.3389/fphar.2021.785403)

Pterostilbene alleviates cholestatic liver injury in a cholestasis mouse model by promoting SIRT1 activity in hepatocytes and macrophages

Chuanrui Ma[1,5,#], Guangyan Yang[2,#], Jiaqing Xiang[2,#], Xinyu Wang[2], Han Wu[2], Zhen Liang[2], Lin Kang[2,4], Shu Yang[2,3,*]

Supplementary figures (the whole uncropped images of the original western blots)

Figure 3E SIRT1

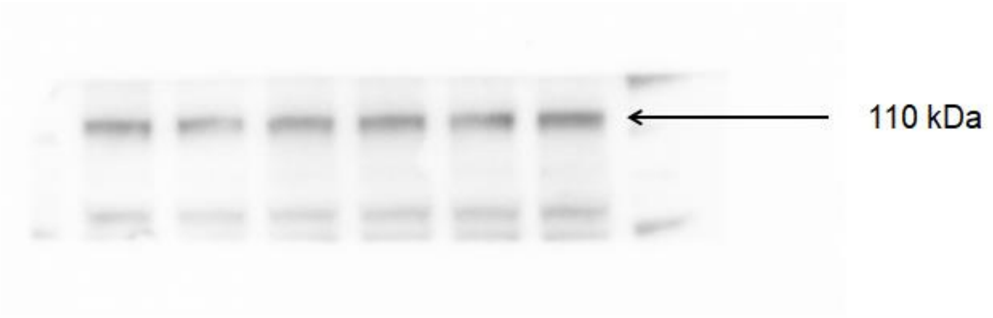

Figure 3E p19Arf

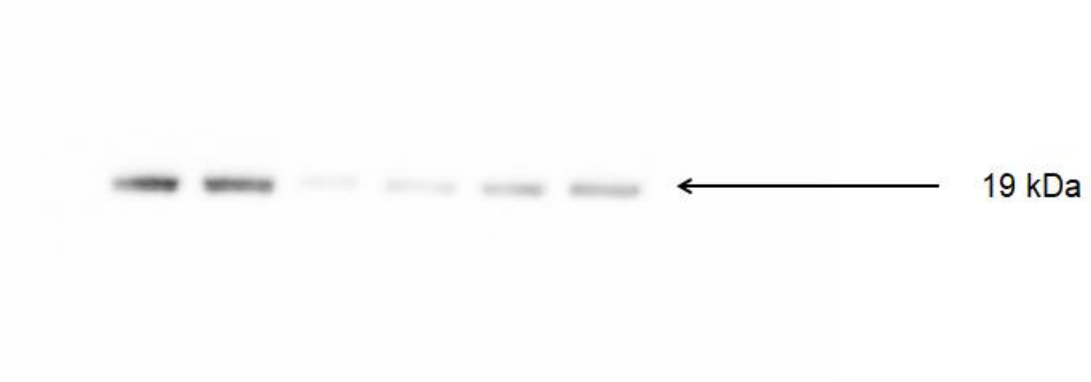

Figure 3E p53

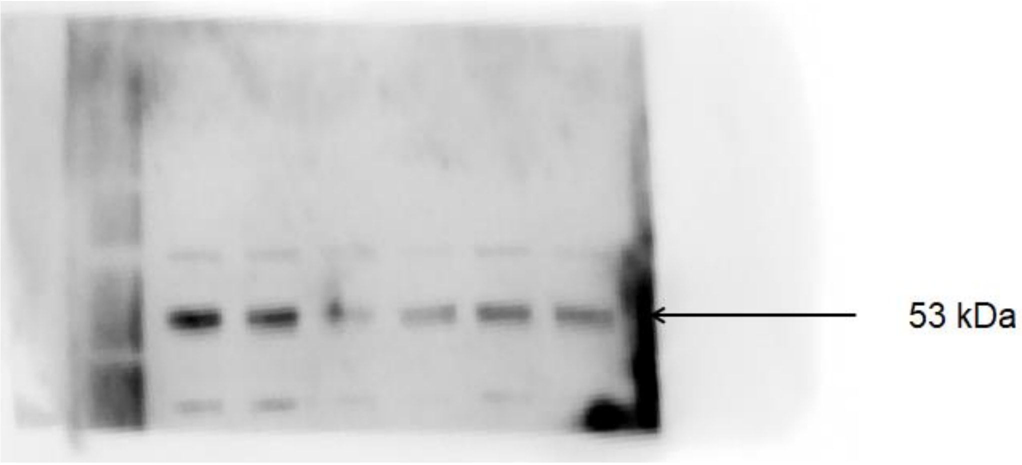

Figure 3E Ub-p53

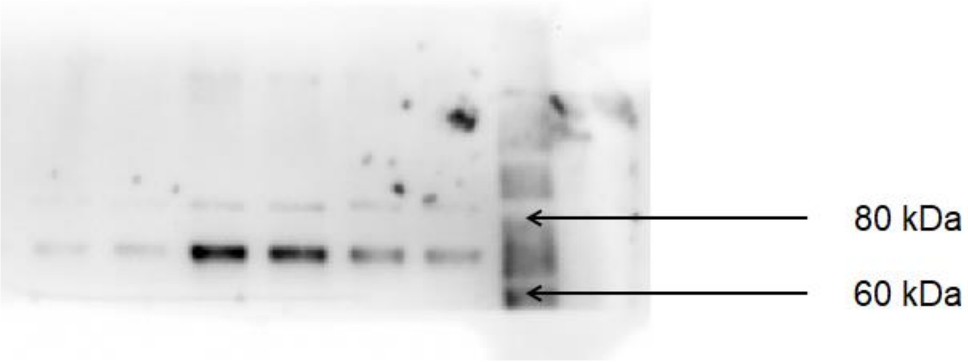

Figure 3E ACTB

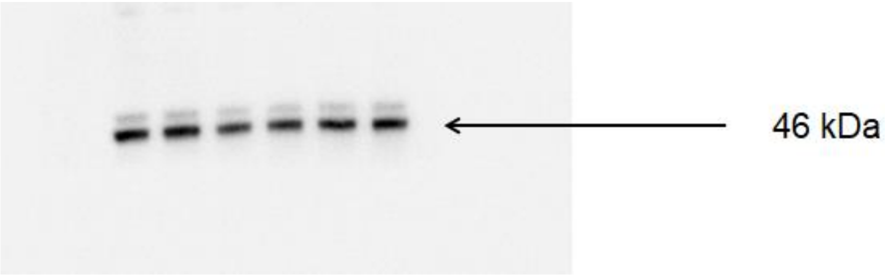

Figure 4A Ac-FXR

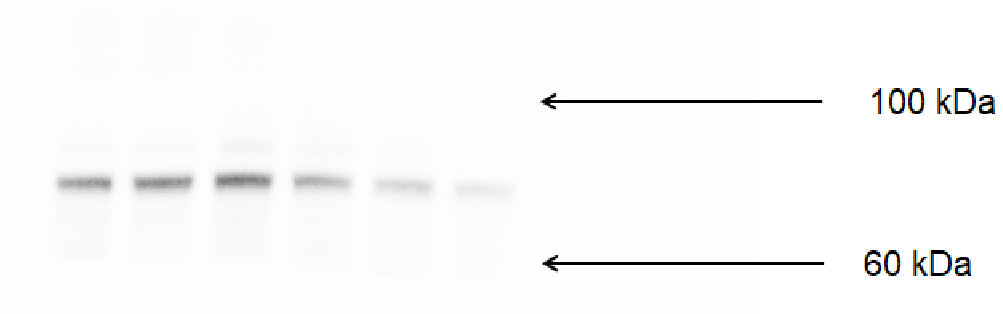

Figure 4A Input-FXR

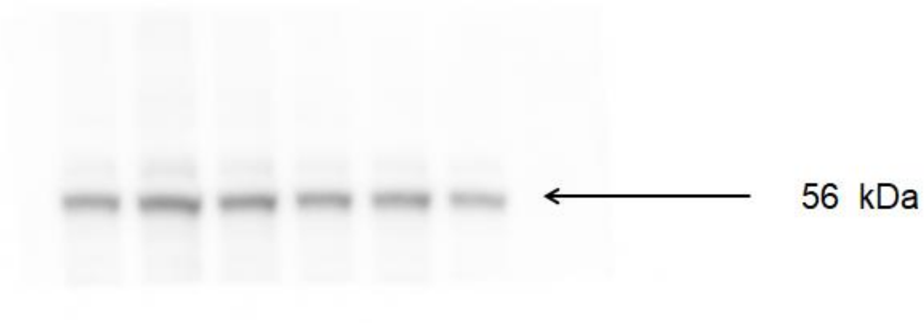

Figure 4B FXR

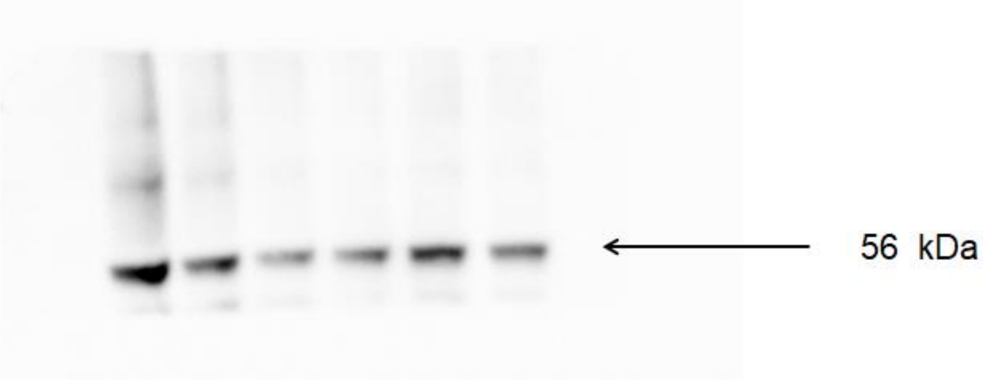

Figure 4B ACTB

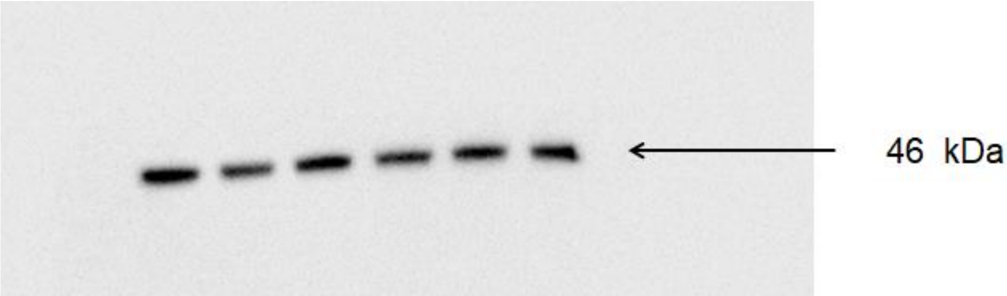

Figure 4F Ac-FXR

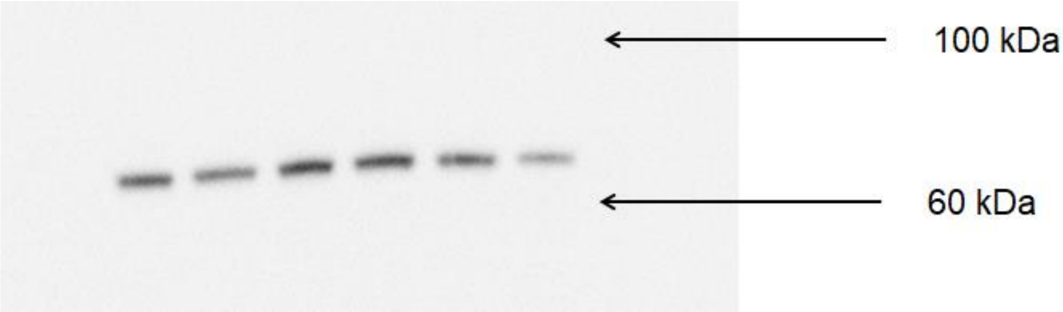

Figure 4F Input-FXR

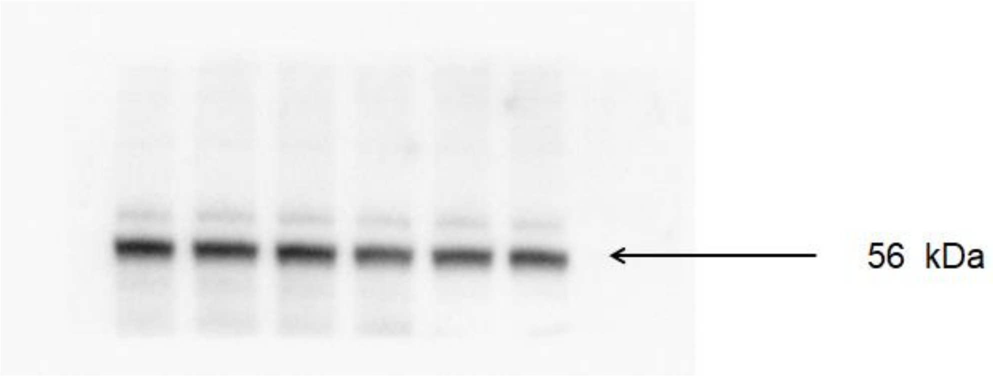

Figure 4G FXR

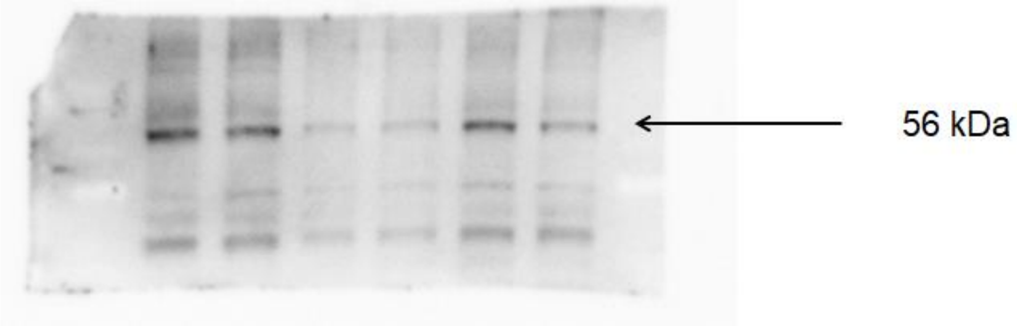

Figure 4G ACTB

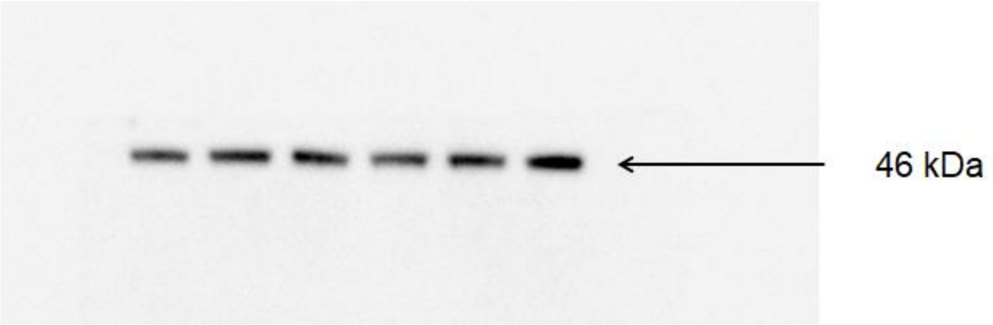

Figure 5C Ac-FXR

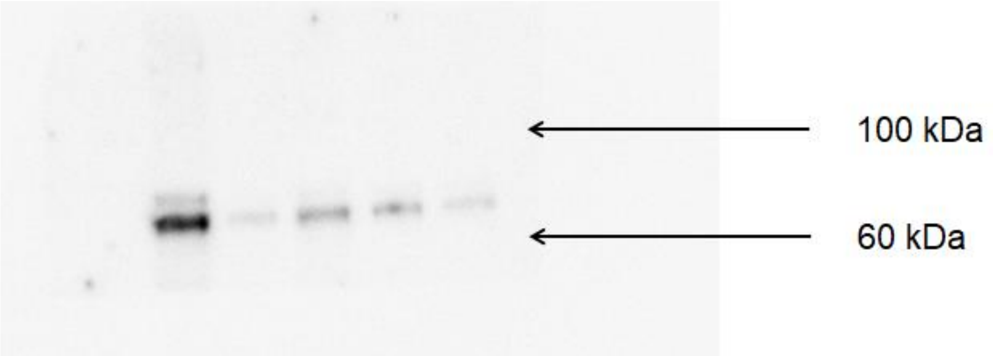

Figure 5C ACTB

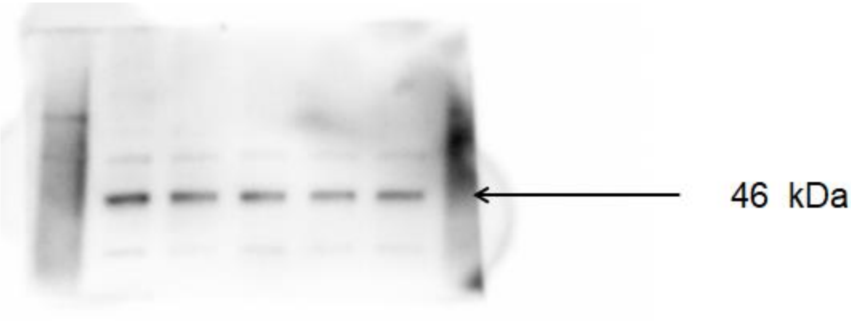

Figure 5D ACTB

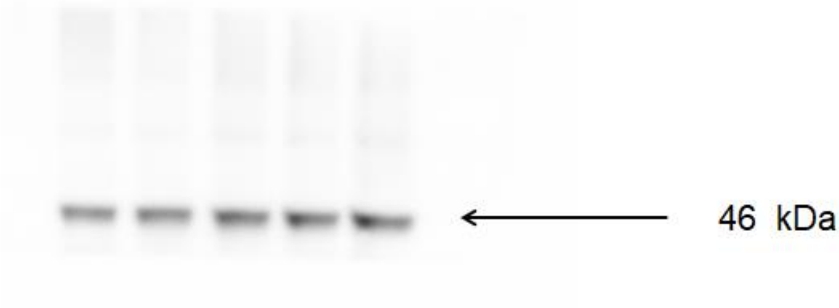

Figure 5D FXR

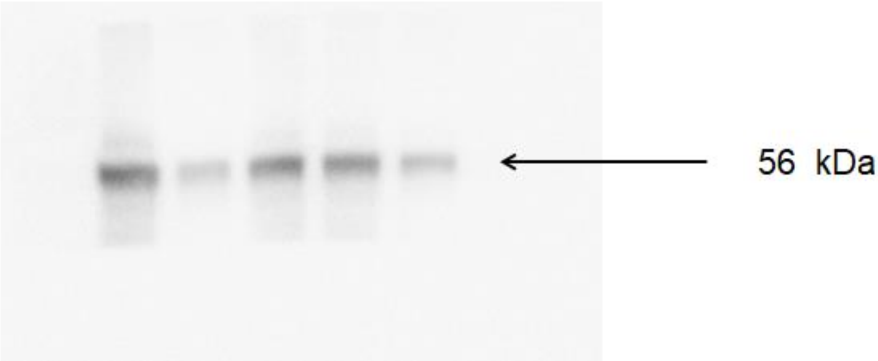

Figure 5E Input-FXR

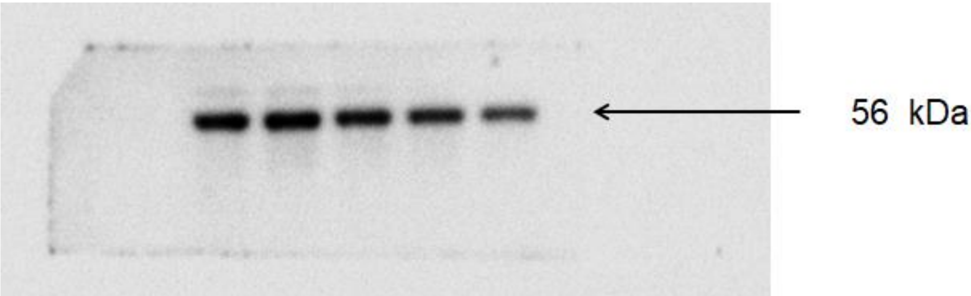

Figure 5E Ac-FXR

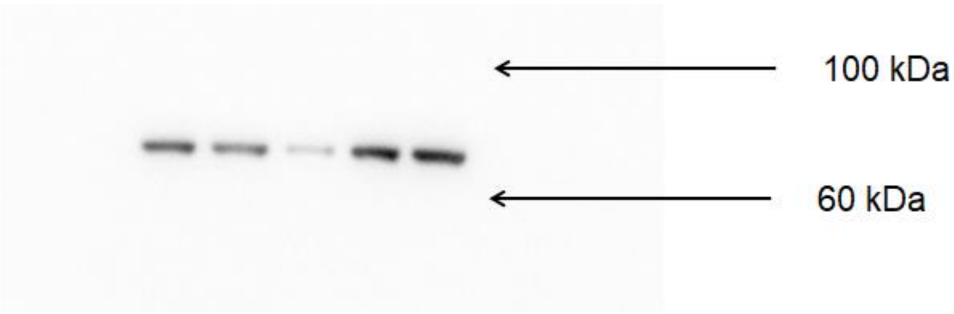

Figure 5F FXR

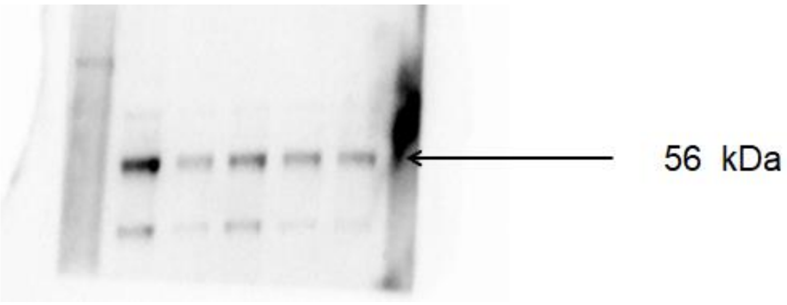

Figure 5F SIRT1

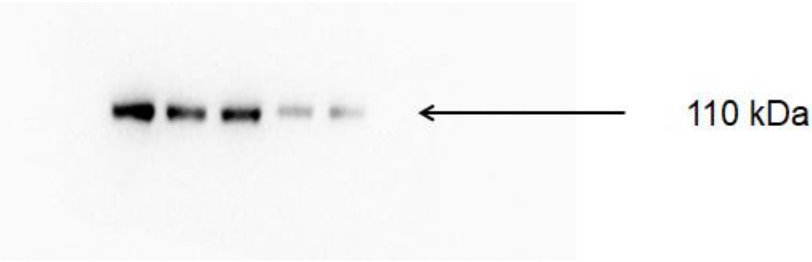

Figure 5F ACTB

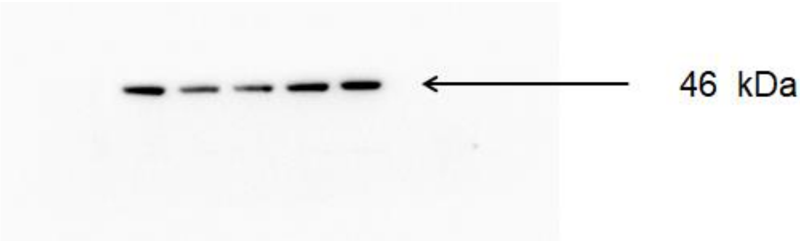

Figure 6A Ub-p53

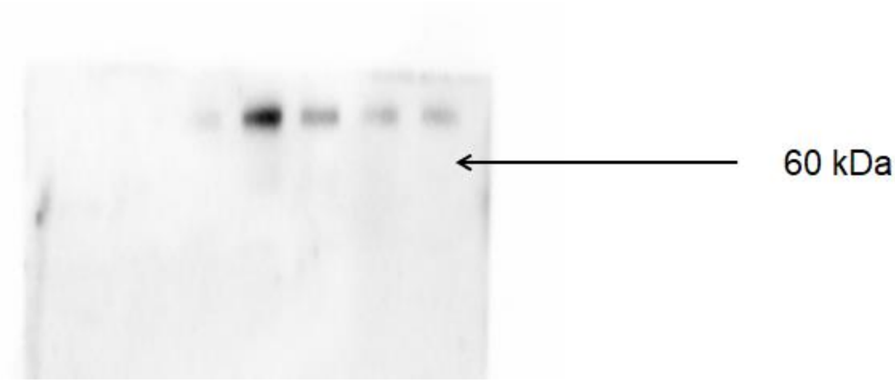

Figure 6A nonUb-p53

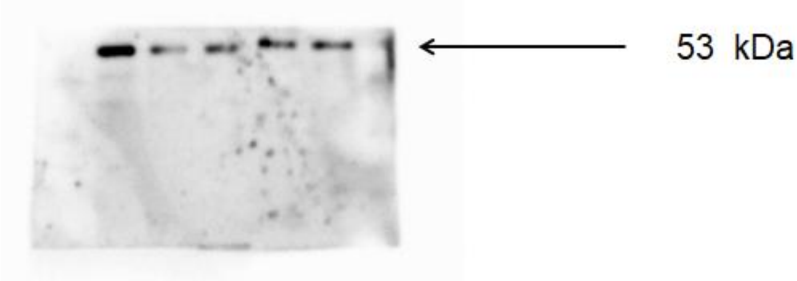

Figure 6B SIRT1

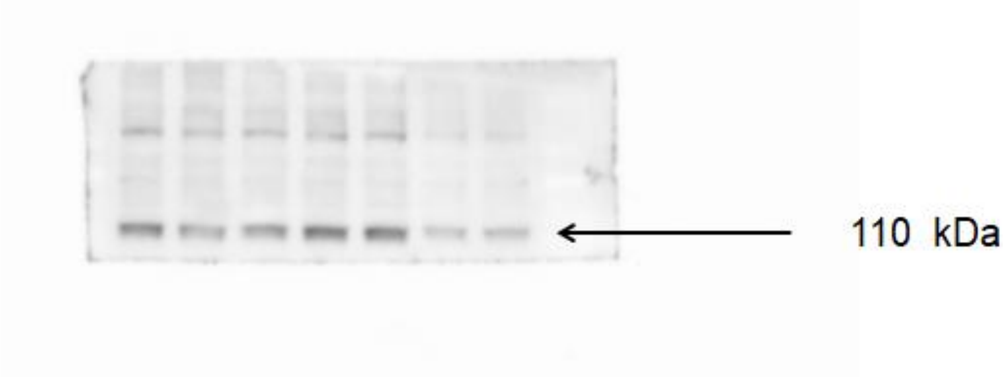

Figure 6B p53

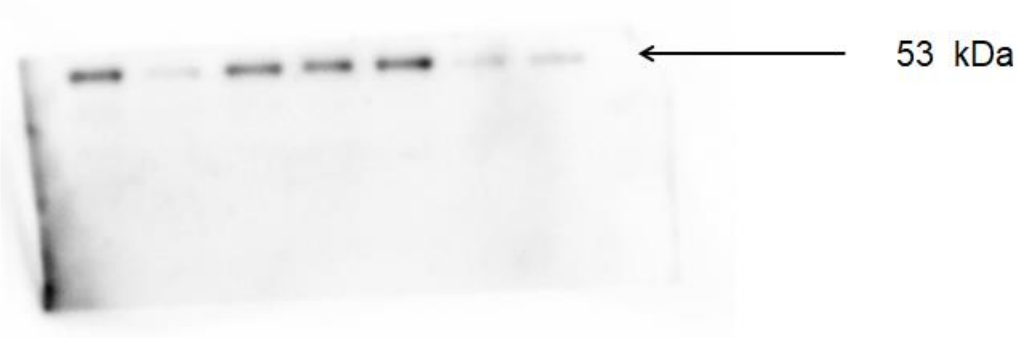

Figure 6B PUMA

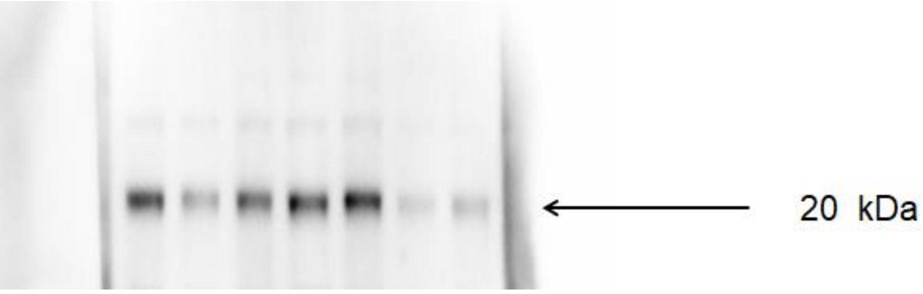

Figure 6B p-IKBA

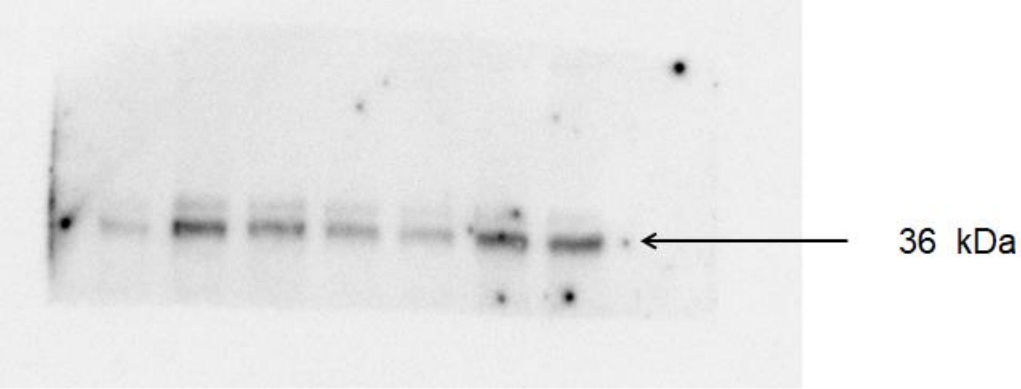

Figure 6B IKBA

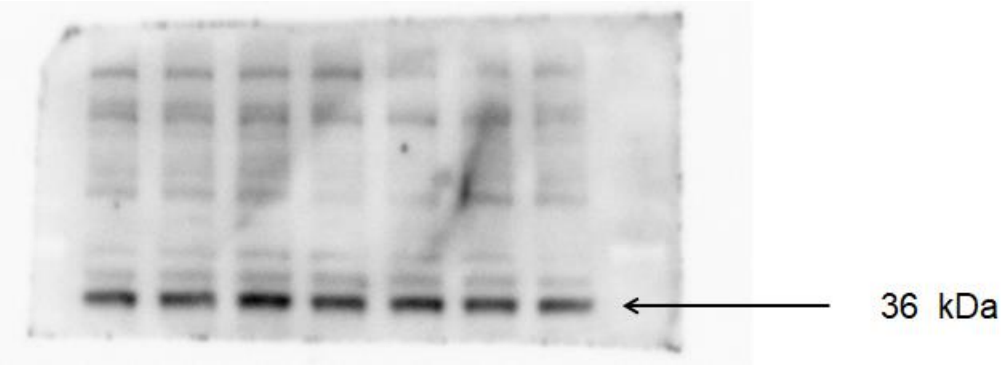

Figure 6B p19Arf

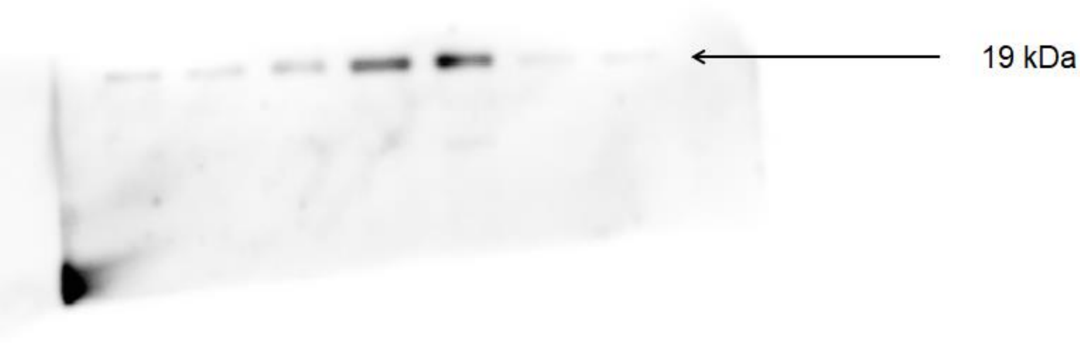

Figure 6B ACTB

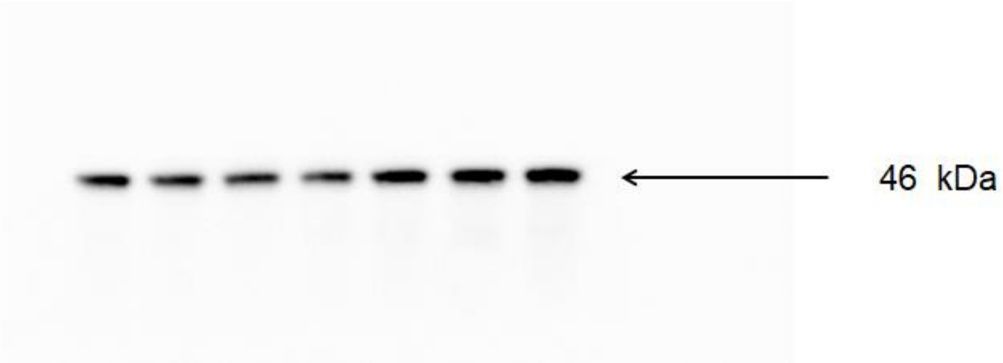

Supplement: Supplementary file 1 [file DataSheet1.PDF]
