# Supplementary material for: Pterostilbene Alleviates Cholestasis by Promoting SIRT1 Activity in Hepatocytes and Macrophages
Source: Front Pharmacol. 2021 Nov 25;12:785403. doi: 10.3389/fphar.2021.785403 (PMC8656168; doi:10.3389/fphar.2021.785403)
Supplement: Supplementary file 2 [file DataSheet2.docx]

**Supporting information for**

**Pterostilbene alleviates cholestasis by promoting SIRT1 activity in hepatocytes and macrophages**

Running title: Pterostilbene alleviates intrahepatic cholestasis by enhancing SIRT1 activity

Chuanrui Ma^1,5,6^, Guangyan Yang^2^, Jiaqing Xiang^2^, Xinyu Wang^2^, Han Wu^2^, Zhen Liang^2^, Lin Kang^2,4^, Shu Yang^2,3,*^

^1^First Teaching Hospital of Tianjin University of Traditional Chinese Medicine, Tianjin, China;

^2^Department of Geriatrics, Shenzhen People’s Hospital (The Second Clinical Medical College, Jinan University; The First Affiliated Hospital, Southern University of Science and Technology), Shenzhen 518020, China;

^3^Integrated Chinese and Western Medicine Postdoctoral Research Station, Jinan University, Guangzhou 510632, China;

^4^The Biobank of National Innovation Center for Advanced Medical Devices, Shenzhen People’s Hospital, Shenzhen 518020, Guangdong, China

^5^Tianjin Key Laboratory of Translational Research of TCM Prescription and Syndrome, Tianjin, China;

^6^National Clinical Research Center for Chinese Medicine Acupuncture and Moxibustion

Correspondence should be addressed to:

Shu Yang, PhD

Department of Endocrinology

The Second Clinical Medical College, Jinan University (Shenzhen People's Hospital), Shenzhen 518020, China;

Tel: 86-18579069720; Email: yang.shu@szhospital.com

**Supporting figures and figure legends**


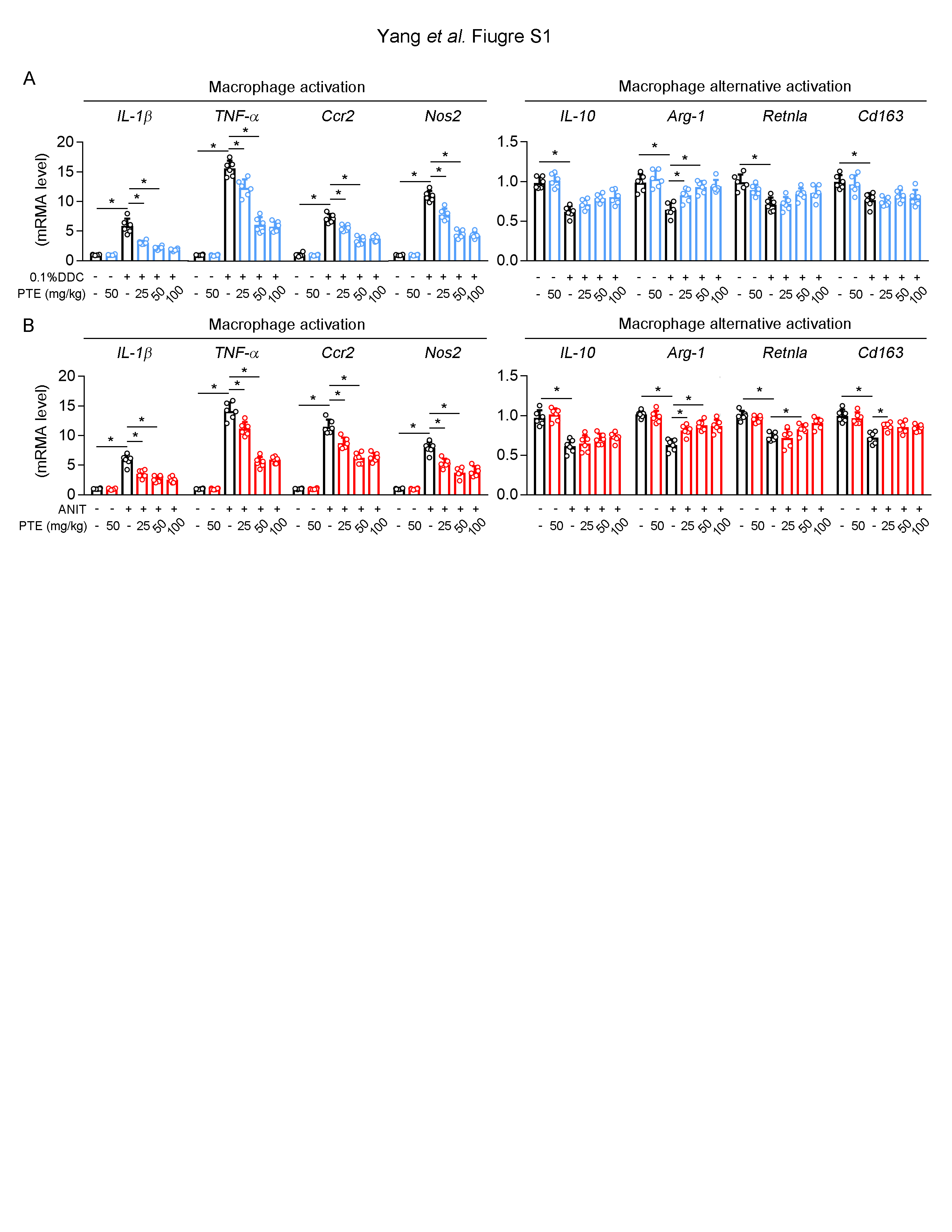


**Figure S1. PTE treatment inhibited macrophage activation genes expression and mildly increased macrophage alternative activation genes expression. (A)** qPCR analysis of *IL-1b, TNF-a, Ccr2, Nos2, IL-10, Arg-1, Retnla,* and *Cd163* in the liver of mice treated with DDC, n=6. **(B)** qPCR analysis of *IL-1b, TNF-a, Ccr2, Nos2, IL-10, Arg-1, Retnla,* and *Cd163* in the liver of mice fed with ANIT, n=6. *p<0.05. The data represent the mean ± SD.
